# Long‐Term Blood Pressure Variability and Physical Performance in Older Adults

**DOI:** 10.1111/jch.70139

**Published:** 2025-09-17

**Authors:** Kerry M. Sheets, Katherine L. Webb, Robyn L. Woods, Suzanne G. Orchard, Lawrence Beilin, Michelle A. Fravel, Christopher M. Reid, Kevan R. Polkinghorne, Rory Wolfe, Zhen Zhou, Joanne Ryan, Anne M. Murray, Michael E. Ernst

**Affiliations:** ^1^ Department of Medicine Hennepin Healthcare Minneapolis Minnesota USA; ^2^ Department of Medicine University of Minnesota Minneapolis Minnesota USA; ^3^ School of Public Health and Preventive Medicine Monash University Melbourne Victoria Australia; ^4^ Medical School Royal Perth Hospital University of Western Australia Perth Western Australia Australia; ^5^ Department of Pharmacy Practice and Science College of Pharmacy The University of Iowa Iowa City Iowa USA; ^6^ School of Population Health Curtin University Perth Western Australia Australia; ^7^ Department of Nephrology Monash Health Clayton Victoria Australia; ^8^ Berman Center for Outcomes and Clinical Research Hennepin Health Research Institute Minneapolis Minnesota USA; ^9^ Department of Family and Community Medicine Carver College of Medicine The University of Iowa Iowa City Iowa USA

**Keywords:** blood pressure variability, gait speed, grip strength, physical performance

## Abstract

High variability in long‐term blood pressure (BPV) independently predicts cardiovascular disease and cognitive decline. Increased BPV and declining physical performance may share mechanistic pathways. However, associations of BPV with gait speed and grip strength have not been examined. We completed a gender‐stratified analysis of 16 692 participants enrolled in ASPREE/ASPREE‐XT. Systolic and diastolic BPV were estimated from baseline‐year 2 (Y2); gait speed/grip strength were assessed every 1–2 years following this period. Linear mixed models examined gait speed/grip strength trajectories over a median of 7.3 years of follow‐up after Y2. Following adjustment, men with SBPV in tertile 3 (T3) versus T1 had slower gait speed at Y2 (0.021 m/s slower) and greater declines in gait speed (0.003 m/s greater decline/year, *p* < 0.001). Women with SBPV in T3 versus T1 had slower gait speed at Y2 (0.018 m/s slower), but similar rates of gait speed decline. Men with higher SBPV had weaker grip strength at Y2 (0.994 kg weaker for BPV T3 vs. T1) and greater declines in grip strength (0.016 kg greater decline/year/5 mmHg increase in BPV, *p* = 0.006). Women with BPV in T3 versus T1 had 0.486 kg weaker grip strength at Y2, but similar rates of grip strength decline. Associations of DBPV and SBPV with gait speed/grip strength were largely consistent. In summary, we found that higher BPV was independently associated with slower gait speed and weaker grip strength cross‐sectionally in men and women, but only associated with trajectories of gait speed and grip strength in men. Future studies should examine high BPV as a target to preserve physical performance.

**Trial Registration**: ISRCTN number: ISRCTN83772183; ClinicalTrials.gov identifier: NCT01038583

## 1 Introduction

Increased visit‐to‐visit, or long‐term, blood pressure variability (BPV) has emerged as an important risk factor for adverse aging‐related outcomes, including cardiovascular disease [1, 2, 3, 4, 5], frailty [6, 7, 8, 9], and mortality [1, 2, 3, 4, 5], independent of mean blood pressure. Long‐term BPV considers the dynamic nature of blood pressure and quantifies the degree of fluctuation in blood pressure over months to years. Physiologically, lower long‐term BPV is associated with preserved arterial compliance and appropriate baroreflex and endothelial function [10, 11, 12]. Long‐term BPV may be modifiable through excellent antihypertensive medication adherence [10, 13, 14], use of specific classes of antihypertensive medications such as calcium channel blockers [15, 16], and healthy lifestyle habits including regular physical activity [17].

Measures of physical performance, such as gait speed and grip strength, provide important information about physical function in older adults and are independently predictive of adverse aging‐related outcomes, including loss of independence [18, 19, 20] and mortality [20, 21]. Gait speed and grip strength are influenced by a complex combination of modifiable and nonmodifiable components, including anthropometric factors, chronic medical conditions, lifestyle factors, and brain health [22, 23, 24]. There may be shared mechanistic pathways between increased BPV and declines in physical performance. For example, lower long‐term BPV is associated with lower burden of cerebrovascular small vessel disease (CSVD) [25] and lower burden of CSVD has been associated with preserved gait speed in cross‐sectional analyses [26, 27]. Since blood pressure is measured routinely in practice, identification of a relationship between higher BPV and physical performance measures could improve risk stratification of older adults; however, it is unclear whether BPV is associated with physical performance.

In addition, it is unknown if any associations of BPV with physical performance vary by gender. Long‐term BPV is higher in women compared to men, and BPV may be a stronger determinant of longevity in women compared to men [28], but lower BPV may be more protective against dementia and cognitive decline in men than in women [29]. Longevity is greater in women compared with men, but poor physical performance and functional dependence are more common in women [30].

Our objective in this post‐hoc analysis was to determine gender‐specific associations of long‐term systolic and diastolic BPV with gait speed and grip strength among 16 692 comprehensively phenotyped women and men aged 65 years and older, enrolled in the ASPirin in Reducing Events in the Elderly (ASPREE) trial and ASPREE—eXTension (ASPREE‐XT), its post‐trial observational follow‐up. As a condition of enrollment, all ASPREE participants were without cardiovascular disease, dementia, or persistent physical disability at baseline. We quantified associations of long‐term BPV with trajectories of gait speed and grip strength and assessed the extent to which long‐term BPV is independently predictive of gait speed and grip strength trajectories.

## 2 Methods

### 2.1 Study Design and Participants

This was a post hoc exploratory analysis of data from ASPREE [31] and ASPREE‐XT [32]. The ASPREE trial (2010–2017) was a placebo‐controlled randomized trial of low‐dose aspirin (100 mg) which enrolled 19 114 community‐dwelling older adults aged 70 years and older (aged 65 years and older for US Black and Hispanic older adults) in the United States and Australia between 2010 and 2014. Key exclusion criteria included a history of cardiovascular disease, uncontrolled hypertension (systolic blood pressure [SBP] ≥180 mmHg and/or diastolic blood pressure [DBP] ≥105 mmHg), persistent physical disability (severe difficulty or inability to independently perform at least one of the following: bathing, transferring from chair to bed, toileting, dressing, eating, or walking across a room), dementia, or any severe illness with remaining life expectancy of <5 years. The trial and observational extension were approved by local institutional review boards at each site, and all participants provided written informed consent. Approximately 83% of participants consented to ongoing observational follow‐up under ASPREE‐XT (2018–2024) with up to 13 years of longitudinal follow‐up for participants enrolled in both ASPREE and ASPREE‐XT. Of the 19 114 participants enrolled in ASPREE, our analytic cohort consisted of 7376 men and 9316 women with annual blood pressure measurements at the baseline through to Year 2 (Y2) study visits during the ASPREE clinical trial period and assessment of gait speed and grip strength at one or more follow‐up study visits beginning from Y2 through ASPREE/ASPREE‐XT (Figures S1 and S2).

### 2.2 Long‐Term BPV

Three standardized blood pressure measurements were collected at each ASPREE annual visit using a validated oscillometric monitor in accordance with American Heart Association guidelines [33]. The average of the three readings was considered the blood pressure for that visit. Multiple indices have been used to estimate BPV; however, there is no universal consensus on the best measure for quantification of long‐term BPV [12]. In prior ASPREE analyses, BPV indices derived using four different methods (standard deviation [SD] of mean SBP, average real variability [ARV], coefficient of variation, and variation independent of the mean) were found to be highly correlated with Pearson's correlation coefficients of 0.89–0.99 [1]. Consistent with these prior studies [1, 29] and for ease of interpretation, we quantified long‐term systolic BPV using the within‐individual SD of the mean systolic blood pressures obtained at each of the baseline, Y1, and Y2 study visits for our primary analysis. ARV was least correlated with SD in a prior ASPREE analysis [1], and was, therefore, used to quantify systolic BPV in sensitivity analyses.

We also explored the relationship between diastolic BPV and physical performance. Consistent with the primary systolic BPV analyses, we estimated diastolic BPV as the within‐individual SD of the mean diastolic blood pressures obtained at each of the baseline, Y1, and Y2 study visits.

### 2.3 Gait Speed and Grip Strength

Gait speed and grip strength were measured biennially during the ASPREE trial (baseline, Y2, Y4, Y6, and 2017 close‐out visits) and annually during ASPREE‐XT [34]. For gait speed, participants walked at their usual pace over a 3‐m course on an indoor, flat surface with at least 1 m available at the end of the walking course to maintain their usual pace over the full course. If unable to walk unassisted, participants were allowed to use an assistive device. Gait speed was measured twice, and the mean of the two measurements was calculated as the visit gait speed. Grip strength was measured using a Jamar hydraulic hand grip dynamometer in accordance with the American Society of Hand Therapists protocol [35]. Grip strength in kilograms was tested one hand at a time for a maximum of three trials per hand, with a 15‐to‐20 s rest between each trial. The mean of the three measurements for each hand was recorded as the visit grip strength, with the strongest hand measurement used in analyses.

### 2.4 Statistical Analysis

Analyses were a priori stratified by gender. Linear mixed effects models were used to determine the association between BPV and trajectories of gait speed and grip strength, with follow‐up starting at Y2 (end of the BPV estimation window, Figure S2). The models included a random intercept for each individual and fixed effects for time (visit number was the time variable), BPV (estimate of the cross‐sectional associations of BPV with gait speed or grip strength at Y2, our model baseline), and a time‐BPV interaction (estimate of the associations of BPV with longitudinal trajectories of gait speed or grip strength). Additional covariates were added as fixed effects. We considered age, race and ethnicity, years of education, height, abdominal circumference, diabetes, depression, statin use, smoking status, dyslipidemia, and 3MS score at baseline; aspirin randomization group; and average systolic blood pressure from baseline‐Y2 in adjusted SPBV analyses and average diastolic blood pressure from baseline‐Y2 in adjusted DBPV analyses.

Multiple pre‐specified sensitivity analyses were performed to assess the robustness of our results. First, ARV was used to estimate BPV using the mean SBP from the baseline, Y1, and Y2 study visits. Second, we extended the BPV estimation window to include systolic blood pressure from the third annual visit (extended four visit BPV estimation window: baseline, Y1, Y2, and Y3). Third, we excluded participants who reported use of an assistive device (walking aid) to ambulate at baseline from gait speed analyses.

In an exploratory analysis, we stratified analyses by both gender and hypertension status at baseline. Hypertension was defined as a blood pressure of ≥140/90 or the use of an antihypertensive medication at baseline.

Statistical analyses were performed using R version 4.3.3 (R Core Team, 2024).

## 3 Results

Our analytic cohort consisted of 7376 men and 9316 women (mean age at baseline of 75 years for both men and women) (Table 1). Mean systolic BPV ranged from 4.23 mmHg in tertile 1 [T1] to 16.60 mmHg in tertile 3 [T3] in men and from 4.44 mmHg in T1 to 17.36 mmHg in T3 in women. From the lowest to the highest tertile of systolic BPV among both men and women, participants had higher baseline SBP and DBP, higher rates of utilization of antihypertensive medications, and more frequent chronic kidney disease (CKD). Among men, rates of smoking (current or past smoker) and diabetes increased, and mean grip strength decreased with increasing systolic BPV.

**TABLE 1:** Participant characteristics at baseline, by gender and systolic BPV tertile.

	Men (*n* = 7376)				Women (*n* = 9316)			
	Overall	Tertile 1	Tertile 2	Tertile 3	Overall	Tertile 1	Tertile 2	Tertile 3
Age, years, mean (SD)	74.91 (4.36)	74.51 (4.07)	74.84 (4.28)	75.39 (4.67)	75.09 (4.46)	74.69 (4.25)	75.02 (4.36)	75.57 (4.73)
Ethno‐racial group, *n* (%)								
Australian White	6572 (89.1)	2249 (89.4)	2159 (89.5)	2164 (88.4)	7894 (84.7)	2667 (85.3)	2624 (84.8)	2603 (84.0)
US White	309 (4.2)	115 (4.6)	103 (4.3)	91 (3.7)	645 (6.9)	208 (6.7)	226 (7.3)	211 (6.8)
African‐American	213 (2.9)	72 (2.9)	67 (2.8)	74 (3.0)	445 (4.8)	132 (4.2)	144 (4.7)	169 (5.5)
Hispanic/Latino	156 (2.1)	52 (2.1)	42 (1.7)	62 (2.5)	220 (2.4)	83 (2.7)	67 (2.2)	70 (2.3)
Other	126 (1.7)	29 (1.2)	41 (1.7)	56 (2.3)	112 (1.2)	35 (1.1)	32 (1.0)	45 (1.5)
Education years, *n* (%)								
<12	3197 (43.3)	1105 (43.9)	1042 (43.2)	1050 (42.9)	4290 (46.0)	1461 (46.8)	1390 (44.9)	1439 (46.4)
12+	4179 (56.7)	1412 (56.1)	1370 (56.8)	1397 (57.1)	5026 (54.0)	1664 (53.2)	1703 (55.1)	1659 (53.6)
Lives alone, *n* (%)	1470 (19.9)	483 (19.2)	464 (19.2)	523 (21.4)	3855 (41.4)	1248 (39.9)	1281 (41.4)	1326 (42.8)
Current or past smoker, *n* (%)	4153 (56.3)	1380 (54.8)	1336 (55.4)	1437 (58.7)	3183 (34.2)	1073 (34.3)	1044 (33.8)	1066 (34.4)
Body Mass Index, kg/m^2^, mean (SD)	27.98 (3.90)	27.92 (3.86)	27.94 (3.88)	28.09 (3.97)	28.17 (5.20)	28.06 (4.99)	28.05 (5.23)	28.39 (5.35)
Abdominal circumference, cm, mean (SD)	102.01 (10.77)	101.73 (10.71)	102.02 (10.78)	102.27 (10.81)	93.23 (12.87)	93.01 (12.54)	92.95 (12.94)	93.73 (13.13)
Diabetes^a^, *n* (%)	895 (12.1)	267 (10.6)	293 (12.1)	335 (13.7)	828 (8.9)	271 (8.7)	249 (8.1)	308 (9.9)
Depression^b^, *n* (%)	540 (7.3)	180 (7.2)	178 (7.4)	182 (7.4)	1030 (11.1)	361 (11.6)	321 (10.4)	348 (11.2)
Dyslipidemia^c^, *n* (%)	4073 (55.2)	1394 (55.4)	1333 (55.3)	1346 (55.0)	6821 (73.2)	2286 (73.2)	2244 (72.4)	2295 (74.1)
Chronic Kidney Disease^d^, *n* (%)	1715 (25.1)	509 (21.9)	550 (24.5)	656 (28.8)	2296 (26.4)	708 (24.3)	705 (24.5)	883 (30.6)
Aspirin treatment arm, *n* (%)	3646 (49.4)	1256 (49.9)	1187 (49.2)	1203 (49.2)	4634 (49.7)	1577 (50.5)	1523 (49.2)	1534 (49.5)
Gait speed, m/sec, mean (SD)	1.06 (0.22)	1.08 (0.22)	1.06 (0.21)	1.04 (0.21)	0.99 (0.23)	1.01 (0.22)	1.00 (0.22)	0.97 (0.23)
Slow gait speed^e^, *n* (%)	761 (10.3)	220 (8.7)	247 (10.2)	294 (12.0)	537 (5.8)	136 (4.7)	164 (5.3)	227 (7.3)
Walking aid used, *n* (%)	104 (1.4)	34 (1.4)	31 (1.3)	39 (1.6)	250 (2.7)	66 (2.1)	81 (2.6)	103 (3.3)
Grip strength, kg, mean (SD)	35.09 (8.29)	35.74 (8.03)	35.16 (8.16)	34.35 (8.60)	20.78 (5.77)	20.88 (5.86)	20.96 (5.61)	20.49 (5.82)
Weak grip strength^f^, *n* (%)	2456 (33.3)	753 (29.9)	797 (33.0)	906 (37.0)	3843 (41.3)	1252 (40.1)	1250 (40.4)	1341 (43.3)
Systolic BP, mmHg, mean (SD)	141.10 (15.83)	137.87 (13.78)	140.44 (14.83)	145.06 (17.80)	137.51 (16.78)	133.58 (14.48)	136.46 (15.62)	142.53 (18.69)
Diastolic BP, mmHg, mean (SD)	78.05 (9.55)	77.18 (8.95)	77.83 (9.31)	79.16 (10.26)	76.61 (10.20)	75.32 (9.51)	76.29 (9.80)	78.23 (11.03)
Average SBP over BPV estimation period^g^, mean (SD)	139.62 (13.27)	137.67 (13.37)	139.42 (13.10)	141.83 (13.01)	136.18 (13.96)	133.32 (14.00)	135.60 (13.62)	139.64 (13.51)
Systolic BPV^h^, mean (SD)	9.90 (5.85)	4.23 (1.62)	9.03 (1.36)	16.60 (4.44)	10.38 (6.18)	4.44 (1.70)	9.39 (1.42)	17.36 (4.95)
Antihypertensive medication use, *n* (%)	3603 (48.8)	1123 (44.6)	1098 (45.5)	1382 (56.5)	5101 (54.8)	1526 (48.8)	1679 (54.3)	1896 (61.2)
3MS^i^, mean (SD)	92.90 (4.64)	93.17 (4.50)	92.74 (4.77)	92.80 (4.64)	94.21 (4.32)	94.27 (4.32)	94.19 (4.26)	94.16 (4.39)

Abbreviations: ACR, albumin–creatinine ratio; ASPREE, ASPirin in Reducing Events in the Elderly; BL, baseline; BMI, body mass index; BP, blood pressure; CKD, chronic kidney disease; DBP, diastolic blood pressure; eGFR, estimated glomerular filtration rate; SBP, systolic blood pressure.

^a^ Diabetes defined as a self‐report, fasting glucose ≥126 mg/dL, or receiving pharmacologic treatment for diabetes (regardless of fasting glucose level).

^b^ Depression defined as a 10‐item Center for Epidemiological Studies Depression Scale score of ≥8.

^c^ Dyslipidemia defined as serum cholesterol level of ≥212 mg per deciliter (≥5.5 mmol per liter) in Australia and ≥240 mg per deciliter (≥6.2 mmol per liter) in the United States, low‐density lipoprotein level of >160 mg per deciliter (>4.1 mmol per liter), or use of a cholesterol‐lowering medication.

^d^ Chronic kidney disease defined as estimated glomerular filtration rate <60 mL/min per 1.73 m^2^ or albumin to creatinine ratio ≥3 mg/mmol.

^e^ Gait speed <0.8 m/s for men, <0.6 m/s for women, or use of a walking aid.

^f^ Grip strength <32 kg for men or <20 kg for women.

^g^ Baseline, Y1, and Y2.

^h^ Standard deviation of SBP from baseline, Y1, and Y2.

^i^ Modified Mini‐Mental State Examination. Score ranges from 0 to 100, a score of ≥78 was required for study enrolment.

### 3.1 Associations of Systolic BPV With Gait Speed

**FIGURE 1:**
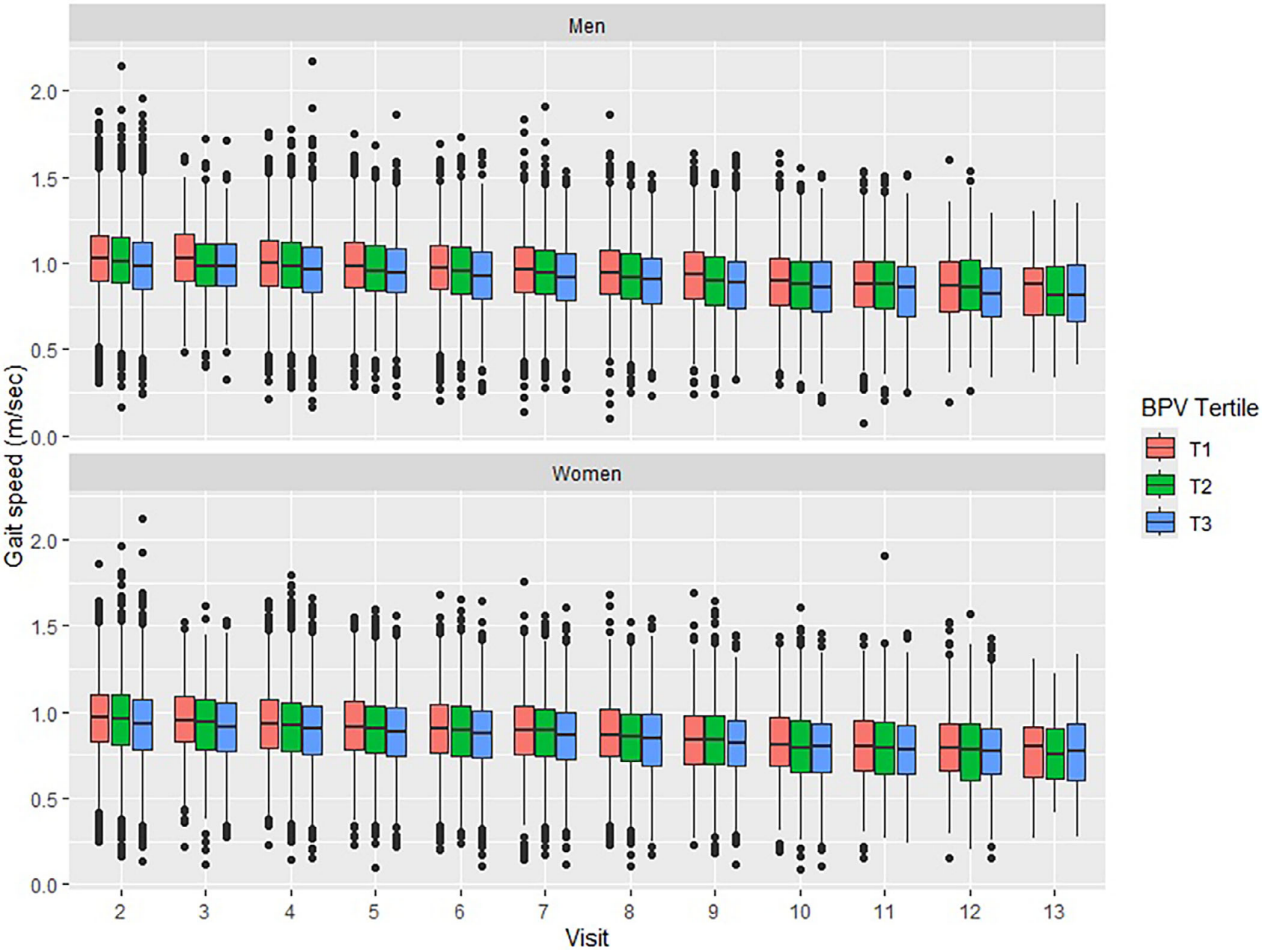
Distribution of gait speed over time, by BPV tertile and gender.

Distributions of gait speed over time by BPV tertile and gender are presented in Figure 1. As expected, gait speed declined in both men and women over a median of 7.3 years of follow‐up after the second annual ASPREE visit (end of the BPV estimation window). Among men, higher systolic BPV was associated cross‐sectionally (at Y2) with slower gait speed and greater decline in gait speed over time in both unadjusted and adjusted models (Table 2). Specifically, men in systolic BPV T3 versus T1 had slower gait speed at Y2 (0.039 m/s slower) and greater decline in gait speed over time (decline of 0.003 m/s more per year) in unadjusted analyses. Following adjustment for demographics, anthropomorphic factors, SBP, and medical and social history (full list of covariates in the Table 2 footnote), the cross‐sectional association between BPV and gait speed was attenuated but remained significant (0.021 m/s slower for men in BPV T3 vs. T1 at Y2; *p* < 0.001), but longitudinal associations were unchanged (decline of 0.003 m/s more per year for men in T3 vs. T1; *p* < 0.001). A similar pattern was observed when BPV was considered as a continuous variable; for each 5 mmHg increase in systolic BPV, gait speed at Y2 was 0.008 m/s slower (*p* < 0.001) and declined 0.001 m/s more per year (*p* < 0.001) in fully adjusted analyses. The relationships between BPV and gait speed trajectories are depicted visually in Figure S3A.

**TABLE 2:** Longitudinal changes in gait speed, by gender.

	Continuous BPV (per 5 mmHg)				T1	T2		T3	
Men, *N*		7376			2517	2412		2447	
BPV range					0–6.66	6.81–11.53		11.55–41.74	

	Time	BPV^a^	Interaction^b^	Time		BPV^a^	Interaction^b^	BPV^a^	Interaction^b^
Unadjusted	−0.021 (*p* < 0.001)	−0.016 (*p* < 0.001)	−0.001 (*p* < 0.001)	−0.022 (*p* < 0.001)	Ref.	−0.014 (*p* = 0.01)	−0.002 (*p* = 0.003)	−0.039 (*p* < 0.001)	−0.003 (*p* < 0.001)
Adjusted^c^	−0.021 (*p* < 0.001)	−0.008 (*p* < 0.001)	−0.001 (*p* < 0.001)	−0.022 (*p* < 0.001)	Ref.	−0.006 (*p* = 0.21)	−0.002 (*p* = 0.003)	−0.021 (*p* < 0.001)	−0.003 (*p* < 0.001)
Women, *N*	9316				3125	3093		3098	
BPV range					0–7.02	7.09–12.06		12.10–57.71	

	Time	BPV^a^	Interaction^b^	Time		BPV^a^	Interaction^b^	BPV^a^	Interaction^b^
Unadjusted	−0.023 (*p* < 0.001)	−0.016 (*p* < 0.001)	0 (*p* = 0.59)	−0.023 (*p* < 0.001)	Ref.	−0.014 (*p* = 0.01)	0 (*p* = 0.35)	−0.040 (*p* < 0.001)	−0.001 (*p* = 0.27)
Adjusted^c^	−0.024 (*p* < 0.001)	−0.007 (*p* < 0.001)	0 (*p* = 0.42)	−0.024 (*p* < 0.001)	Ref.	−0.008 (*p* = 0.07)	0 (*p* = 0.47)	−0.018 (*p* < 0.001)	−0.001 (*p* = 0.20)

^a^ Estimate of the cross‐sectional association of BPV with gait speed at Y2 (BPV was assessed from baseline to Y2, associations of BPV with gait speed are assessed from Y2 to end of follow‐up for each participant).

^b^ Time‐BPV interaction term to test variation of the gait speed trajectory among BPV levels.

^c^ Adjusted for baseline age, race/ethnicity, education, height, abdominal circumference, diabetes, depression, statin use, smoking status, dyslipidemia, and 3MS score; randomized aspirin; and average SBP baseline‐Y2.

Among women, systolic BPV was associated with slower gait speed at Y2 but not a greater decline in gait speed over time. In both unadjusted and adjusted analyses, women in systolic BPV T3 versus T1 had a slower gait speed at Y2 (unadjusted: 0.040 m/s slower; adjusted: 0.018 m/s slower) but similar rates of gait speed decline over time (Time‐BPV interaction of –0.001; *p* > 0.05).

### 3.2 Associations of Systolic BPV With Grip Strength

**FIGURE 2:**
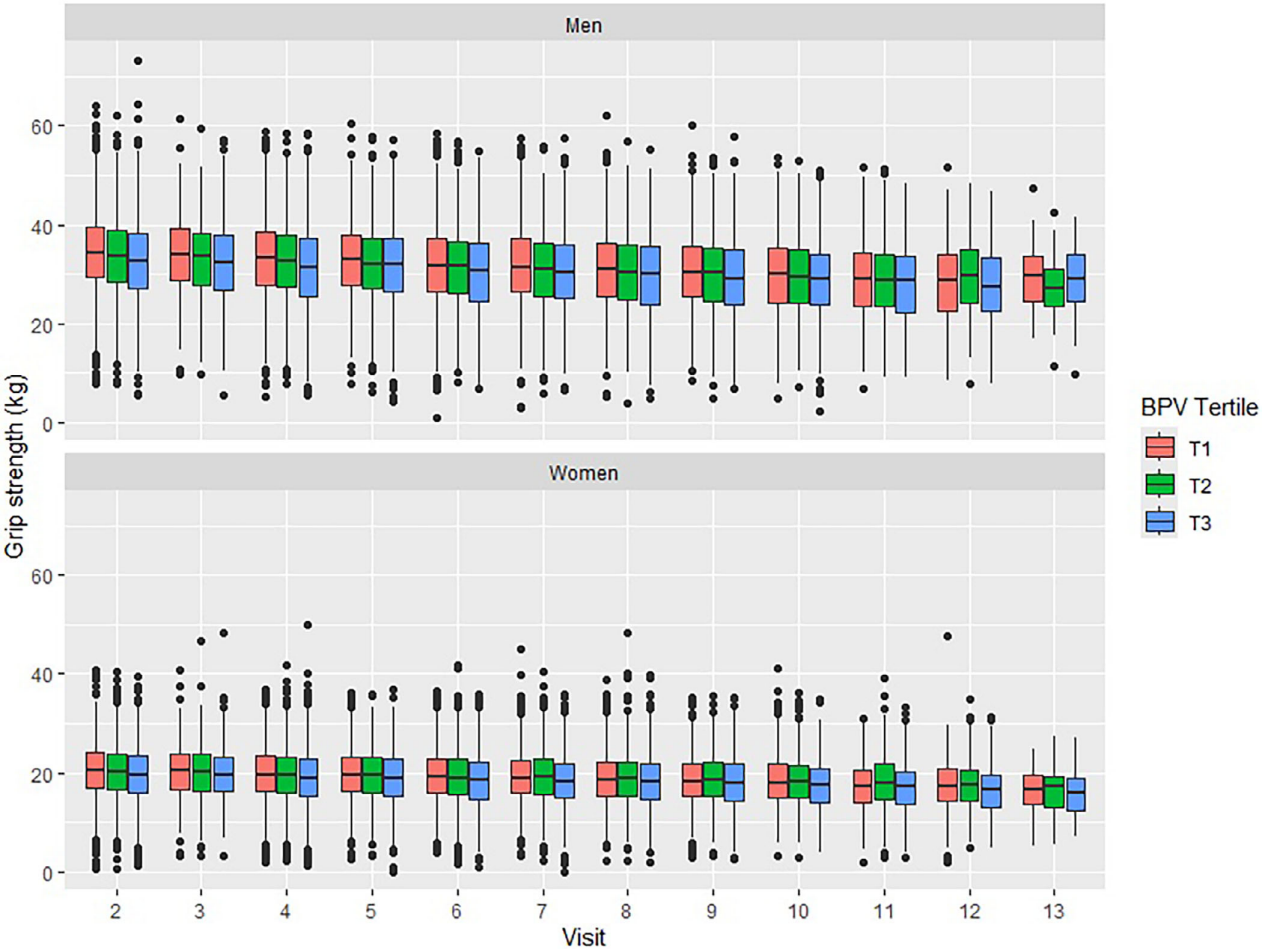
Distribution of grip strength over time, by BPV tertile and gender.

Distributions of grip strength over time by BPV tertile and gender are presented in Figure 2. Similar to gait speed, grip strength declined over time in both men and women. Among men, greater systolic BPV was associated with weaker cross‐sectional grip strength and greater decline in grip strength over time when BPV was considered as a continuous variable in both unadjusted and adjusted analyses (Table 3). Men in systolic BPV T3 versus T1 had weaker grip strength at Y2 (unadjusted: 1.59 kg weaker; adjusted: 0.99 kg weaker). Both the unadjusted and adjusted associations between systolic BPV and grip strength trajectories were nonsignificant in analyses considering tertiles of BPV (adjusted: BPV*time interaction of –0.027, *p* = 0.10). However, when systolic BPV was considered as a continuous variable, for each 5 mmHg increase in systolic BPV, grip strength declined on average 0.016 kg more per year (*p* = 0.006) in both unadjusted and adjusted analyses. The relationships between BPV and grip strength trajectories are depicted visually in Figure 3B.

**TABLE 3:** Longitudinal changes in grip strength, by gender.

	Continuous BPV (per 5 mmHg)				T1	T2		T3	
Men, *N*	7376				2517	2412		2447	
BPV range					0–6.66	6.81–11.53		11.55–41.74	

	Time	BPV^a^	Interaction^b^	Time		BPV^a^	Interaction^b^	BPV^a^	Interaction^b^
Unadjusted	−0.652 (*p* < 0.001)	−0.662 (*p* < 0.001)	−0.016 (*p* = 0.009)	−0.677 (*p* < 0.001)	Ref.	−0.555 (*p* = 0.01)	0.008 (*p* = 0.63)	−1.592 (*p* < 0.001)	−0.025 (*p* = 0.13)
Adjusted^c^	−0.656 (*p* < 0.001)	−0.404 (*p* < 0.001)	−0.016 (*p* = 0.006)	−0.681 (*p* < 0.001)	Ref.	−0.310 (*p* = 0.13)	0.007 (*p* = 0.67)	−0.994 (*p* < 0.001)	−0.027 (*p* = 0.10)
Women, *N*	9316				3125	3093		3098	
BPV range					0–7.02	7.09–12.06		12.10–57.71	

	Time	BPV^a^	Interaction^b^	Time		BPV^a^	Interaction^b^	BPV^a^	Interaction^b^
Unadjusted	−0.336 (*p* < 0.001)	−0.290 (*p* < 0.001)	−0.003 (*p* = 0.36)	−0.343 (*p* < 0.001)	Ref.	−0.170 (*p* = 0.19)	0.010 (*p* = 0.37)	−0.764 (*p* < 0.001)	−0.008 (*p* = 0.47)
Adjusted^c^	−0.339 (*p* < 0.001)	−0.170 (*p* < 0.001)	−0.004 (*p* = 0.30)	−0.348 (*p* < 0.001)	Ref.	−0.091 (*p* = 0.45)	0.011 (*p* = 0.31)	−0.486 (*p* < 0.001)	−0.009 (*p* = 0.44)

^a^ Estimate of the cross‐sectional association of BPV with grip strength at Y2 (BPV was assessed from baseline to Y2, associations of BPV with grip strength are assessed from Y2 to end of follow‐up for each participant).

^b^ Time‐BPV interaction term to test variation of the grip strength trajectory among BPV levels.

^c^ Adjusted for baseline age, race/ethnicity, education, height, abdominal circumference, diabetes, depression, statin use, smoking status, dyslipidemia, and 3MS score; randomized aspirin; and average SBP baseline‐Y2.

Among women, greater systolic BPV was associated with weaker cross‐sectional grip strength, but no greater decline in grip strength over time. In unadjusted analyses, women in systolic BPV T3 versus T1 had weaker grip strength at Y2 (0.76 kg weaker), but no greater decline in grip strength over time (BPV*time interaction of –0.008, *p* = 0.47). The cross‐sectional associations were mildly attenuated following consideration of potential confounders. In fully adjusted analyses, women in systolic BPV T3 versus T1 had 0.49 kg weaker grip strength at Y2, and no greater decline in grip strength over time (BPV*time interaction *p* value of 0.44).

### 3.3 Associations of Diastolic BPV With Gait Speed and Grip Strength

Among both men and women, associations of diastolic BPV with gait speed (Table S1) and grip strength (Table S2) were largely consistent with associations observed with systolic BPV. However, in contrast to the primary systolic BPV analyses, adjusted associations of continuous diastolic BPV with gait speed at baseline and grip strength decline over time were nonsignificant among men.

### 3.4 Sensitivity and Exploratory Analyses

Among both men and women, associations of systolic BPV assessed as ARV and gait speed (Table S3) and grip strength (Table S4) were largely consistent with associations of systolic BPV assessed as SD and gait speed and grip strength. Associations of systolic BPV and gait speed were also largely unchanged in analyses excluding participants who reported use of a walking aid at baseline (Table S5) and in analyses in which the BPV estimation window was extended to include the blood pressure reading from the Y3 visit (Table S6). In grip strength analyses in which the BPV estimation window was extended, the association between systolic BPV considered as a continuous variable and grip strength trajectories was attenuated and no longer significant among men, but the association between BPV and cross‐sectional grip strength gained significance for men with systolic BPV in T2 versus T1 (0.78 kg weaker; Table S7). Associations of BPV estimated using the extended estimation window and grip strength were otherwise largely consistent with associations of BPV estimated through the Y2 visit and grip strength.

Associations of systolic BPV with gait speed (Table S8) and grip strength (Table S9) were largely unchanged among men and women with hypertension at baseline. Associations of systolic BPV with gait speed and grip strength were attenuated and generally no longer significant among men and women without hypertension at baseline.

## 4 Discussion

In this post‐hoc analysis of more than 16 000 initially healthy, community‐dwelling ASPREE participants, we found that higher systolic and diastolic BPV were independently associated with greater declines in gait speed and grip strength over time in older men but not older women. Higher systolic and diastolic BPV were associated cross‐sectionally with slower gait speed and weaker grip strength in both men and women. These associations remained consistent when systolic BPV was quantified as ARV instead of SD and following adjustment for known risk factors for declines in physical performance such as global cognitive function. Overall, our results suggest higher BPV is a risk factor for slower gait speed and weaker grip strength in older adults, particularly among older men.

We are unaware of any prior research examining associations of BPV with gait speed and grip strength. Our results are consistent with the broader BPV literature, which has shown higher BPV is independently associated with increased risk for adverse aging‐related outcomes including cardiovascular disease [1, 2, 3, 4, 5], frailty [6, 7, 8, 9], need for assistance with daily activities [36], and mortality [1, 2, 3, 4, 5]. Gait speed is a complex motor function influenced by a diverse range of modifiable and nonmodifiable factors through multiple separate, but interconnected, physiologic pathways [22, 23, 24, 37]. Our results suggest higher BPV may be one of the pathophysiologic mechanisms that contribute to slow gait speed in older adults. One possible mechanism is through the impact of BPV on CSVD. Higher long‐term BPV is associated with a greater burden of CSVD [25], and a greater burden of CSVD is cross‐sectionally associated with slower gait speed [26, 27]. Another possible mechanism is through the hypothesized effects of BPV on the larger vasculature in the brain; higher long‐term BPV is a risk factor for ischemic stroke [1], and stroke has been found to be a risk factor for slow gait speed in some analyses [22, 37]. Alternatively, higher BPV may serve as a marker, but not a direct cause, of underlying physiologic changes associated with declines in gait speed. For example, higher BPV may serve as a marker of homeostatic disruption [6]. Slow gait speed is a hypothesized manifestation of severe homeostatic dysfunction [38], and increases in BPV may precede declines in gait speed in the presence of developing homeostatic dysfunction.

Grip strength has traditionally been conceptualized as a marker of overall muscle strength [39, 40], but is also hypothesized to be influenced by neural systems that control coordinated movement [41]. Weak grip strength is a key marker of sarcopenia, a muscle disease characterized by low muscle strength and low muscle quantity or quality [42]. Sarcopenia was associated with higher BPV in a small study of older adults with diabetes [43]. Higher BPV and weak grip strength may be connected through shared risk factors; for example, physical inactivity is associated with both higher BPV [17] and the development of sarcopenia [42]. Higher BPV may also be mechanistically related to the development of weak grip strength. For example, a greater burden of white matter hyperintensities, a manifestation of CSVD, is associated with lower muscle mass [44], and higher long‐term BPV is associated with a greater burden of CSVD [25]. The neural systems that control coordinated movement are also detrimentally impacted by CSVD [41].

We found that higher BPV was independently associated with slower gait speed and weaker grip strength cross‐sectionally in both men and women, but was only associated with trajectories of gait speed and grip strength in men. Gender‐based differences have also been observed in prior BPV analyses. For example, visit‐to‐visit BPV over one year was more strongly associated with subsequent mortality in women compared to men enrolled in the US Cohort of the International Verapamil SR‐Trandolapril Study [28]. Conversely, higher long‐term BPV was more strongly associated with subsequent development of dementia in men compared to women in a prior ASPREE analysis [29]. In fact, gender‐based differences have been observed in relation to a variety of blood pressure parameters and subsequent outcomes. For example, risk for cardiovascular disease increased at a lower SBP threshold in women than in men in an analysis of over 27 000 participants enrolled in four community‐based cohort studies [45]. An analysis of hypertension and gait speed in the Cardiovascular Health Study (CHS) found that declines in gait speed were greater in men with hypertension compared to women with hypertension [46]. Exposure to sex hormones such as estrogens and androgens may play a role in the observed sex‐based differences. Sex hormones affect vascular function [47, 48], and sex is considered a key biological variable in cardiovascular research [47, 49]. Future research is needed in additional cohorts with detailed information on lifetime exposure to sex hormones to confirm and improve understanding of our hypothesis‐generating finding of sex‐based differences in the impact of long‐term BPV on physical performance.

In an exploratory analysis, we found that higher BPV may be a more significant risk factor among older adults with hypertension compared to those without. However, three‐quarters of participants met criteria for hypertension at baseline (BP ≥140/90 or use of antihypertensive medication), and thus it is unclear whether the observed differences were due to effect modification or insufficient power given the smaller sample of participants without hypertension. Studies of associations of BPV with CVD [1, 3] have had mixed results regarding the potential for effect modification by hypertension status.

With respect to clinical significance, a within‐individual change in gait speed of 0.1–0.2 m/s [50] and a within‐individual change in grip strength of 5.0–6.5 kg [51] are thought to be clinically‐significant. The magnitude of the longitudinal differences we observed (decline in gait speed of 0.002 m/s more per year in men with systolic BPV in T3 versus T1, decline in grip strength of 0.016 kg more per year in men per 5 mmHg increase in systolic BPV) may be more relevant at a population level than an individual level. The longitudinal differences in gait speed trajectories among men with BPV in T3 versus T1 are somewhat smaller in magnitude compared to differences observed in older adults with hypertension versus without hypertension in a longitudinal analysis of data from CHS (decline in gait speed of 0.005–0.007 m/s more per year in older adults with vs. without hypertension) [46]. The authors of the CHS analysis note that the associations of hypertension with declines in gait speed are most relevant when considered over the 13 years of follow‐up used in the analysis [46]. Our results can be interpreted in a comparable manner—the impact of higher BPV on gait speed and grip strength likely becomes more clinically important (on the individual level) over time.

While gait speed and grip strength are highly predictive of aging‐related outcomes, they are not routinely measured in clinical practice settings. As such, tools like the electronic health record (EHR) frailty index (eFI) leverage data already available in the EHR to predict important aging‐related outcomes such as falls, subsequent healthcare utilization, and mortality [52]. Blood pressure is routinely measured in clinical care settings and could be leveraged to calculate long‐term BPV. While future study is needed, our results suggest inclusion of long‐term BPV in risk prediction models has the potential to improve identification of older adults at risk for declines in physical performance and function.

Our study has strengths including its prospective design, comprehensive phenotyping of over 16 000 participants with over 7 years of follow‐up, consideration of both systolic BPV and diastolic BPV, and inclusion of participants with and without hypertension. However, there are limitations. There is the potential for unmeasured confounding with the observational analytic design. There is the potential for bias through non‐biologic factors that affect BPV such as poor medication adherence. For example, physical performance measures are strongly correlated with functional independence and dependence [18, 19, 20], and thus non‐biologic factors such as poor medication adherence have the potential to be most common among participants with slow gait speed and weak grip strength. Our analysis did not separately consider the effects of uncontrolled hypertension, as >60% of participants with hypertension had BP ≥140/90 mmHg at the baseline visit (results not shown). Due to the timing of medication data (collected at annual visits), we were also unable to account for the effect of interval changes in antihypertensive regimens. Of importance, >99% of participants who were taking antihypertensive medications at baseline remained on antihypertensive therapy for the duration of the ASPREE trial [1] and less than 3% had a change (addition or deletion of medication) in antihypertensive medications across the visits used to estimate BPV (results not shown). Gait speed was measured over 3 m, as opposed to the more standard 4 m, although all results were analyzed in meters per second, and gait speed over 2.44 m (8 feet) and gait speed over 4 m have been shown to be highly correlated [53]. With respect to the observed gender‐based differences, we were unable to account for the age of menopause or lifetime estrogen exposure among women. Finally, our results are associative, and only data from a prospective clinical trial can determine whether specifically targeting BPV in older adults can lead to slower declines in gait speed and grip strength.

In conclusion, we found that higher BPV was independently associated with greater declines in gait speed and grip strength over time in men and slower cross‐sectional gait speed and weaker cross‐sectional grip strength in both men and women. Our results suggest higher BPV is a candidate variable for inclusion in disability risk assessment in older adults, and should be further explored as a potentially modifiable risk factor for decline in physical performance in older adults.

## Ethics Statement

The trial and observational extension were approved by local institutional review boards at the study sites. All participants provided written informed consent.

## Conflicts of Interest

The authors declare no conflicts of interest.

## Supporting information




**Supplemental Figure 1**: Participant Flow Diagram.
**Supplemental Figure 2**: BPV estimation and outcome ascertainment periods.
**Supplemental Figure 3**: Predicted gait speed and grip strength from fully adjusted model, by gender and BPV tertile. (A) Gait Speed, (B) Grip Strength.
**Supplemental Table 1**: Longitudinal changes in gait speed, by gender, with BPV calculated using diastolic blood pressure.
**Supplemental Table 2**: Longitudinal changes in grip strength, by gender, with BPV calculated using diastolic blood pressure.
**Supplemental Table 3**: Longitudinal changes in gait speed, by gender, with BPV calculated using ARV (average real variability).
**Supplemental Table 4**: Longitudinal changes in grip strength, by gender, with BPV calculated using ARV (average real variability).
**Supplemental Table 5**: Longitudinal changes in gait speed, by gender; excluding participants who reported use of a walking aid at baseline.
**Supplemental Table 6**: Longitudinal changes in gait speed, by gender, with long‐term blood pressure variability calculated using four blood pressure measurements (from baseline to the year 3 visit).
**Supplemental Table 7**: Longitudinal changes in grip strength, by gender, with long‐term blood pressure variability calculated using four blood pressure measurements (from baseline to the year 3 visit).
**Supplemental Table 8**: Longitudinal changes in gait speed, by gender and hypertension status at baseline.
**Supplemental Table 9**: Longitudinal changes in grip strength, by gender and hypertension status at baseline.

## Data Availability

ASPREE data are available to partnering and external researchers through the ASPREE website (https://aspree.org/usa/).

## References

[1] M. E. Ernst , E. K. Chowdhury , L. J. Beilin , et al., “Long‐Term Blood Pressure Variability and Risk of Cardiovascular Disease Events Among Community‐Dwelling Elderly,” Hypertension 76, no. 6 (2020): 1945–1952, 10.1161/HYPERTENSIONAHA.120.16209. 33131315 PMC7666049

[2] C. Wu , M. G. Shlipak , R. S. Stawski , et al., “Visit‐to‐Visit Blood Pressure Variability and Mortality and Cardiovascular Outcomes Among Older Adults: The Health, Aging, and Body Composition Study,” American Journal of Hypertension 30, no. 2 (2017): 151–158, 10.1093/ajh/hpw106. 27600581 PMC5225946

[3] E. Y. F. Wan , E. Y. T. Yu , W. Y. Chin , D. Y. T. Fong , E. P. H. Choi , and C. L. K. Lam , “Association of Visit‐to‐Visit Variability of Systolic Blood Pressure With Cardiovascular Disease, Chronic Kidney Disease and Mortality in Patients With Hypertension,” Journal of Hypertension 38, no. 5 (2020): 943–953, 10.1097/HJH.0000000000002347. 31904623

[4] S. L. Stevens , S. Wood , C. Koshiaris , et al., “Blood Pressure Variability and Cardiovascular Disease: Systematic Review and Meta‐Analysis,” British Medical Journal 354 (2016): i4098, 10.1136/bmj.i4098. 27511067 PMC4979357

[5] P. Muntner , J. Whittle , A. I. Lynch , et al., “Visit‐to‐Visit Variability of Blood Pressure and Coronary Heart Disease, Stroke, Heart Failure, and Mortality: A Cohort Study,” Annals of Internal Medicine 163, no. 5 (2015): 329–338, 10.7326/M14-2803. 26215765 PMC5021508

[6] M. A. Fravel , M. E. Ernst , R. L. Woods , et al., “Long‐Term Blood Pressure Variability and Frailty Risk in Older Adults,” Journal of Hypertension 42, no. 2 (2024): 244–251, 10.1097/HJH.0000000000003599. 38009310 PMC10842997

[7] Y. Xu , Q. Lv , Y. Liu , et al., “Association of Long‐Term Blood Pressure With Frailty Progression in Older Adults: A Prospective Cohort Study,” American Journal of Preventive Medicine 69, no. 4 (2025): 107735, 10.1016/j.amepre.2025.107735. 40451343

[8] T. Zanotto , T. H. Mercer , A. Gupta , M. L. van der Linden , and P. Koufaki , “Blood Pressure Variability and Frailty in End‐Stage Kidney Disease,” Journal of Frailty & Aging 13, no. 4 (2024): 534–540, doi:10.14283/jfa.2024.61. 39574279

[9] L. Rouch , S. De , P. Barreto , O. Hanon , et al., “Visit‐to‐Visit Blood Pressure Variability and Incident Frailty in Older Adults,” Journals of Gerontology. Series A, Biological Sciences and Medical Sciences 76, no. 8 (2021): 1369–1375, 10.1093/gerona/glab112. 33844014

[10] A. B. Sheikh , P. A. Sobotka , I. Garg , et al., “Blood Pressure Variability in Clinical Practice: Past, Present and the Future,” Journal of the American Heart Association 12, no. 9 (2023): e029297, 10.1161/JAHA.122.029297. 37119077 PMC10227216

[11] D. Shimbo , S. Shea , R. L. McClelland , et al., “Associations of Aortic Distensibility and Arterial Elasticity With Long‐Term Visit‐to‐Visit Blood Pressure Variability: The Multi‐Ethnic Study of Atherosclerosis (MESA),” American Journal of Hypertension 26, no. 7 (2013): 896–902, 10.1093/ajh/hpt040. 23537891 PMC3693480

[12] G. Parati , G. Bilo , A. Kollias , et al., “Blood Pressure Variability: Methodological Aspects, Clinical Relevance and Practical Indications for Management—A European Society of Hypertension Position Paper^∗^ ,” Journal of Hypertension 41, no. 4 (2023): 527–544, 10.1097/HJH.0000000000003363. 36723481

[13] I. M. Kronish , A. I. Lynch , S. Oparil , et al., “The Association Between Antihypertensive Medication Nonadherence and Visit‐to‐Visit Variability of Blood Pressure: Findings From the Antihypertensive and Lipid‐Lowering Treatment to Prevent Heart Attack Trial,” Hypertension 68, no. 1 (2016): 39–45, 10.1161/HYPERTENSIONAHA.115.06960. 27217410 PMC4900942

[14] K. Hong , P. Muntner , I. Kronish , D. Shilane , and T. I. Chang , “Medication Adherence and Visit‐to‐Visit Variability of Systolic Blood Pressure in African Americans With Chronic Kidney Disease in the AASK Trial,” Journal of Human Hypertension 30, no. 1 (2016): 73–78, 10.1038/jhh.2015.26. 25833706 PMC4592365

[15] A. de Havenon , N. Petersen , Z. Wolcott , et al., “Effect of Dihydropyridine Calcium Channel Blockers on Blood Pressure Variability in the SPRINT Trial: A Treatment Effects Approach,” Journal of Hypertension 40, no. 3 (2022): 462–469, 10.1097/HJH.0000000000003033. 34694261 PMC11284837

[16] A. J. S. Webb , U. Fischer , Z. Mehta , and P. M. Rothwell , “Effects of Antihypertensive‐Drug Class on Interindividual Variation in Blood Pressure and Risk of Stroke: A Systematic Review and Meta‐Analysis,” Lancet 375, no. 9718 (2010): 906–915, 10.1016/S0140-6736(10)60235-8. 20226989

[17] X. Xu , X. Meng , and S. I. Oka , “Long‐Term Habitual Vigorous Physical Activity Is Associated With Lower Visit‐to‐Visit Systolic Blood Pressure Variability: Insights From the SPRINT Trial,” American Journal of Hypertension 34, no. 5 (2021): 463–466, 10.1093/ajh/hpaa198. 33245323

[18] J. T. Neumann , L. T. P. Thao , A. M. Murray , et al., “Prediction of Disability‐Free Survival in Healthy Older People,” Geroscience 44, no. 3 (2022): 1641–1655, 10.1007/s11357-022-00547-x. 35420334 PMC9213595

[19] N. M. Peel , L. J. Alapatt , L. V. Jones , and R. E. Hubbard , “The Association Between Gait Speed and Cognitive Status in Community‐Dwelling Older People: A Systematic Review and Meta‐Analysis,” Journals of Gerontology. Series A, Biological Sciences and Medical Sciences 74, no. 6 (2019): 943–948, 10.1093/gerona/gly140. 29917045

[20] G. Abellan van Kan , Y. Rolland , S. Andrieu , et al., “Gait Speed at Usual Pace as a Predictor of Adverse Outcomes in Community‐Dwelling Older People an International Academy on Nutrition and Aging (IANA) Task Force,” Journal of Nutrition, Health and Aging 13, no. 10 (2009): 881–889, 10.1007/s12603-009-0246-z.19924348

[21] R. Cooper , D. Kuh , R. Hardy , Mortality Review Group , and FALCon and HALCyon Study Teams , “Objectively Measured Physical Capability Levels and Mortality: Systematic Review and Meta‐Analysis,” British Medical Journal 341 (2010): c4467, 10.1136/bmj.c4467. 20829298 PMC2938886

[22] E. Figgins , F. Pieruccini‐Faria , M. Speechley , and M. Montero‐Odasso , “Potentially Modifiable Risk Factors for Slow Gait in Community‐Dwelling Older Adults: A Systematic Review,” Ageing Research Reviews 66 (2021): 101253, 10.1016/j.arr.2020.101253. 33429086

[23] C. de Araújo Amaral , T. L. M. Amaral , G. T. R. Monteiro , M. T. L. de Vasconcellos , and M. C. Portela , “Factors Associated With Low Handgrip Strength in Older People: Data of the Study of Chronic Diseases (Edoc‐I),” BMC Public Health 20, no. 1 (2020): 395, 10.1186/s12889-020-08504-z. 32216788 PMC7098144

[24] P. J. Pan , C. H. Lin , N. P. Yang , et al., “Normative Data and Associated Factors of Hand Grip Strength Among Elderly Individuals: The Yilan Study, Taiwan,” Scientific Reports 10, no. 1 (2020): 6611, 10.1038/s41598-020-63713-1. 32313118 PMC7170913

[25] Y. Ma , P. Yilmaz , D. Bos , et al., “Blood Pressure Variation and Subclinical Brain Disease,” Journal of the American College of Cardiology 75, no. 19 (2020): 2387–2399, 10.1016/j.jacc.2020.03.043. 32408975 PMC9049233

[26] K. F. de Laat , A. G. W. van Norden , R. A. R. Gons , et al., “Gait in Elderly With Cerebral Small Vessel Disease,” Stroke; A Journal of Cerebral Circulation 41, no. 8 (2010): 1652–1658, 10.1161/STROKEAHA.110.583229. 20576951

[27] B. L. Rosario , A. L. Rosso , H. J. Aizenstein , et al., “Cerebral White Matter and Slow Gait: Contribution of Hyperintensities and Normal‐Appearing Parenchyma,” Journals of Gerontology. Series A, Biological Sciences and Medical Sciences 71, no. 7 (2016): 968–973, 10.1093/gerona/glv224. 26755683 PMC4906323

[28] O. Dasa , S. M. Smith , G. Howard , et al., “Association of 1‐Year Blood Pressure Variability With Long‐Term Mortality Among Adults With Coronary Artery Disease: A Post Hoc Analysis of a Randomized Clinical Trial,” JAMA Network Open 4, no. 4 (2021): e218418, 10.1001/jamanetworkopen.2021.8418. 33914047 PMC8085725

[29] M. E. Ernst , J. Ryan , E. K. Chowdhury , et al., “Long‐Term Blood Pressure Variability and Risk of Cognitive Decline and Dementia Among Older Adults,” Journal of the American Heart Association 10, no. 13 (2021): e019613, 10.1161/JAHA.120.019613. 34176293 PMC8403315

[30] M. V. Zunzunegui , B. E. Alvarado , R. Guerra , et al., “The Mobility Gap Between Older Men and Women: The Embodiment of Gender,” Archives of Gerontology and Geriatrics 61, no. 2 (2015): 140–148, 10.1016/j.archger.2015.06.005. 26113021

[31] ASPREE Investigator Group , “Study Design of ASPirin in Reducing Events in the Elderly (ASPREE): A Randomized, Controlled Trial,” Contemporary Clinical Trials 36, no. 2 (2013): 555–564, 10.1016/j.cct.2013.09.014. 24113028 PMC3919683

[32] M. E. Ernst , J. C. Broder , R. Wolfe , et al., “Health Characteristics and Aspirin Use in Participants at the Baseline of the ASPirin in Reducing Events in the Elderly – eXTension (ASPREE‐XT) Observational Study,” Contemporary Clinical Trials 130 (2023): 107231, 10.1016/j.cct.2023.107231. 37196887 PMC10330669

[33] T. G. Pickering , J. E. Hall , L. J. Appel , et al., “Recommendations for Blood Pressure Measurement in Humans and Experimental Animals: Part 1: Blood Pressure Measurement in Humans: A Statement for Professionals From the Subcommittee of Professional and Public Education of the American Heart Association Council on High Blood Pressure Research,” Hypertension 45, no. 1 (2005): 142–161, 10.1161/01.HYP.0000150859.47929.8e. 15611362

[34] S. G. Orchard , G. Polekhina , J. Ryan , et al., “Combination of Gait Speed and Grip Strength to Predict Cognitive Decline and Dementia,” Alzheimer's & Dementia: Diagnosis, Assessment & Disease Monitoring (Amsterdam, Netherlands) 14, no. 1 (2022): e12353, 10.1002/dad2.12353. PMC9494608 36187193

[35] E. Fess , Grip Strength, 2nd ed. (American Society of Hand Therapists, 1992).

[36] S. R. Cummings , S. Studenski , and L. Ferrucci , “A Diagnosis of Dismobility–Giving Mobility Clinical Visibility: A Mobility Working Group Recommendation,” Journal of the American Medical Association 311, no. 20 (2014): 2061–2062, 10.1001/jama.2014.3033. 24763978 PMC5012417

[37] E. Figgins , Y. H. Choi , M. Speechley , and M. Montero‐Odasso , “Associations Between Potentially Modifiable and Nonmodifiable Risk Factors and Gait Speed in Middle‐ and Older‐Aged Adults: Results From the Canadian Longitudinal Study on Aging,” Journals of Gerontology. Series A, Biological Sciences and Medical Sciences 76, no. 10 (2021): e253–e263, 10.1093/gerona/glab008. 33420785 PMC8522473

[38] L. P. Fried , A. A. Cohen , Q. L. Xue , J. Walston , K. Bandeen‐Roche , and R. Varadhan , “The Physical Frailty Syndrome as a Transition From Homeostatic Symphony to Cacophony,” Nature Aging 1, no. 1 (2021): 36–46, 10.1038/s43587-020-00017-z. 34476409 PMC8409463

[39] C. Beaudart , Y. Rolland , A. J. Cruz‐Jentoft , et al., “Assessment of Muscle Function and Physical Performance in Daily Clinical Practice : A Position Paper Endorsed by the European Society for Clinical and Economic Aspects of Osteoporosis, Osteoarthritis and Musculoskeletal Diseases (ESCEO),” Calcified Tissue International 105, no. 1 (2019): 1–14, 10.1007/s00223-019-00545-w. 30972475

[40] H. C. Roberts , H. J. Denison , H. J. Martin , et al., “A Review of the Measurement of Grip Strength in Clinical and Epidemiological Studies: Towards a Standardised Approach,” Age and Ageing 40, no. 4 (2011): 423–429, 10.1093/ageing/afr051. 21624928

[41] R. G. Carson , “Get a Grip: Individual Variations in Grip Strength Are a Marker of Brain Health,” Neurobiology of Aging 71 (2018): 189–222, 10.1016/j.neurobiolaging.2018.07.023. 30172220

[42] A. J. Cruz‐Jentoft , G. Bahat , J. Bauer , et al., “Sarcopenia: Revised European Consensus on Definition and Diagnosis,” Age and Ageing 48, no. 1 (2019): 16–31, 10.1093/ageing/afy169. 30312372 PMC6322506

[43] Y. Hashimoto , A. Kaji , R. Sakai , et al., “Sarcopenia Is Associated With Blood Pressure Variability in Older Patients With Type 2 Diabetes: A Cross‐Sectional Study of the KAMOGAWA‐DM Cohort Study,” Geriatrics & Gerontology International 18, no. 9 (2018): 1345–1349, 10.1111/ggi.13487. 30039599

[44] K. Kohara , Y. Okada , M. Ochi , et al., “Muscle Mass Decline, Arterial Stiffness, White Matter Hyperintensity, and Cognitive Impairment: Japan Shimanami Health Promoting Program Study,” Journal of Cachexia, Sarcopenia and Muscle 8, no. 4 (2017): 557–566, 10.1002/jcsm.12195. 28371474 PMC5566649

[45] H. Ji , T. J. Niiranen , F. Rader , et al., “Sex Differences in Blood Pressure Associations With Cardiovascular Outcomes,” Circulation 143, no. 7 (2021): 761–763, 10.1161/CIRCULATIONAHA.120.049360. 33587655 PMC7884079

[46] C. Rosano , W. T. Longstreth , R. Boudreau , et al., “High Blood Pressure Accelerates Gait Slowing in Well‐Functioning Older Adults Over 18‐Years of Follow‐Up,” Journal of the American Geriatrics Society 59, no. 3 (2011): 390–397, 10.1111/j.1532-5415.2010.03282.x. 21391929 PMC3637929

[47] L. S. Robison , O. J. Gannon , A. E. Salinero , and K. L. Zuloaga , “Contributions of Sex to Cerebrovascular Function and Pathology,” Brain Research 1710 (2019): 43–60, 10.1016/j.brainres.2018.12.030. 30580011

[48] A. Iorga , C. M. Cunningham , S. Moazeni , G. Ruffenach , S. Umar , and M. Eghbali , “The Protective Role of Estrogen and Estrogen Receptors in Cardiovascular Disease and the Controversial Use of Estrogen Therapy,” Biology of Sex Differences 8, no. 1 (2017): 33, 10.1186/s13293-017-0152-8. 29065927 PMC5655818

[49] J. A. Clayton and M. D. Gaugh , “Sex as a Biological Variable in Cardiovascular Diseases: JACC Focus Seminar 1/7,” Journal of the American College of Cardiology 79, no. 14 (2022): 1388–1397, 10.1016/j.jacc.2021.10.050. 35393021

[50] R. W. Bohannon and S. S. Glenney , “Minimal Clinically Important Difference for Change in Comfortable Gait Speed of Adults With Pathology: A Systematic Review,” Journal of Evaluation in Clinical Practice 20, no. 4 (2014): 295–300, 10.1111/jep.12158. 24798823

[51] R. W. Bohannon , “Minimal Clinically Important Difference for Grip Strength: A Systematic Review,” Journal of Physical Therapy Science 31, no. 1 (2019): 75–78, 10.1589/jpts.31.75. 30774209 PMC6348186

[52] N. M. Pajewski , K. Lenoir , B. J. Wells , J. D. Williamson , and K. E. Callahan , “Frailty Screening Using the Electronic Health Record Within a Medicare Accountable Care Organization,” Journals of Gerontology. Series A, Biological Sciences and Medical Sciences 74, no. 11 (2019): 1771–1777, 10.1093/gerona/glz017. 30668637 PMC6777083

[53] J. M. Guralnik , L. Ferrucci , C. F. Pieper , et al., “Lower Extremity Function and Subsequent Disability: Consistency Across Studies, Predictive Models, and Value of Gait Speed Alone Compared With the Short Physical Performance Battery,” Journals of Gerontology. Series A, Biological Sciences and Medical Sciences 55, no. 4 (2000): M221–231, 10.1093/gerona/55.4.m221. 10811152 PMC12149745

