# Supplementary material for: Long‐Term Blood Pressure Variability and Physical Performance in Older Adults
Source: J Clin Hypertens (Greenwich). 2025 Sep 17;27(9):e70139. doi: 10.1111/jch.70139 (PMC12442049; doi:10.1111/jch.70139)
Supplement: Supplementary file 1 — Supplemental Figure 1: Participant Flow Diagram. Supplemental Figure 2: BPV estimation and outcome ascertainment periods. Supplemental Figure 3: Predicted gait speed and grip strength from fully adjusted model, by gender and BPV tertile. (A) Gait Speed, (B) Grip Strength. Supplemental Table 1: Longitudinal changes in gait speed, by gender, with BPV calculated using diastolic blood pressure. Supplemental Table 2: Longitudinal changes in grip strength, by gender, with BPV calculated using diastolic blood pressure. Supplemental Table 3: Longitudinal changes in gait speed, by gender, with BPV calculated using ARV (average real variability). Supplemental Table 4: Longitudinal changes in grip strength, by gender, with BPV calculated using ARV (average real variability). Supplemental Table 5: Longitudinal changes in gait speed, by gender; excluding participants who reported use of a walking aid at baseline. Supplemental Table 6: Longitudinal changes in gait speed, by gender, with long‐term blood pressure variability calculated using four blood pressure measurements (from baseline to the year 3 visit). Supplemental Table 7: Longitudinal changes in grip strength, by gender, with long‐term blood pressure variability calculated using four blood pressure measurements (from baseline to the year 3 visit). Supplemental Table 8: Longitudinal changes in gait speed, by gender and hypertension status at baseline. Supplemental Table 9: Longitudinal changes in grip strength, by gender and hypertension status at baseline. [file JCH-27-e70139-s001.docx]

**Supplemental Material**

**Supplemental Figure 1.** Participant Flow Diagram.

**Supplemental Figure 2.** BPV estimation and outcome ascertainment periods.

**Supplemental Figure 3.** Predicted gait speed and grip strength from fully adjusted model, by gender and BPV tertile. (A) Gait Speed, (B) Grip Strength.

**Supplemental Table 1.** Longitudinal changes in gait speed, by gender, with BPV calculated using diastolic blood pressure.

**Supplemental Table 2.** Longitudinal changes in grip strength, by gender, with BPV calculated using diastolic blood pressure.

**Supplemental Table 3.** Longitudinal changes in gait speed, by gender, with BPV calculated using ARV (average real variability).

**Supplemental Table 4.** Longitudinal changes in grip strength, by gender, with BPV calculated using ARV (average real variability).

**Supplemental Table 5.** Longitudinal changes in gait speed, by gender; excluding participants who reported use of a walking aid at baseline.

**Supplemental Table 6.** Longitudinal changes in gait speed, by gender, with long-term blood pressure variability calculated using four blood pressure measurements (from baseline to the year 3 visit).

**Supplemental Table 7.** Longitudinal changes in grip strength, by gender, with long-term blood pressure variability calculated using four blood pressure measurements (from baseline to the year 3 visit).

**Supplemental Table 8.** Longitudinal changes in gait speed, by gender and hypertension status at baseline.

**Supplemental Table 9.** Longitudinal changes in grip strength, by gender and hypertension status at baseline.

**Supplemental Figure 1.** Participant inclusion/exclusion flow chart.

Individuals screened
n = 83,376

ASPREE sample
n = 19,114

Participated in year 2 visit
n = 18,598

BPV available
n = 16,758

Gait speed and grip strength measures available in follow-up
n = 16,692

9,316 women
7,376 men

Gait speed not collected
n = 30

Grip strength not collected
n = 29

Neither measure collected
n = 7

**Supplemental Figure 2.** BPV estimation and outcome ascertainment periods.

**
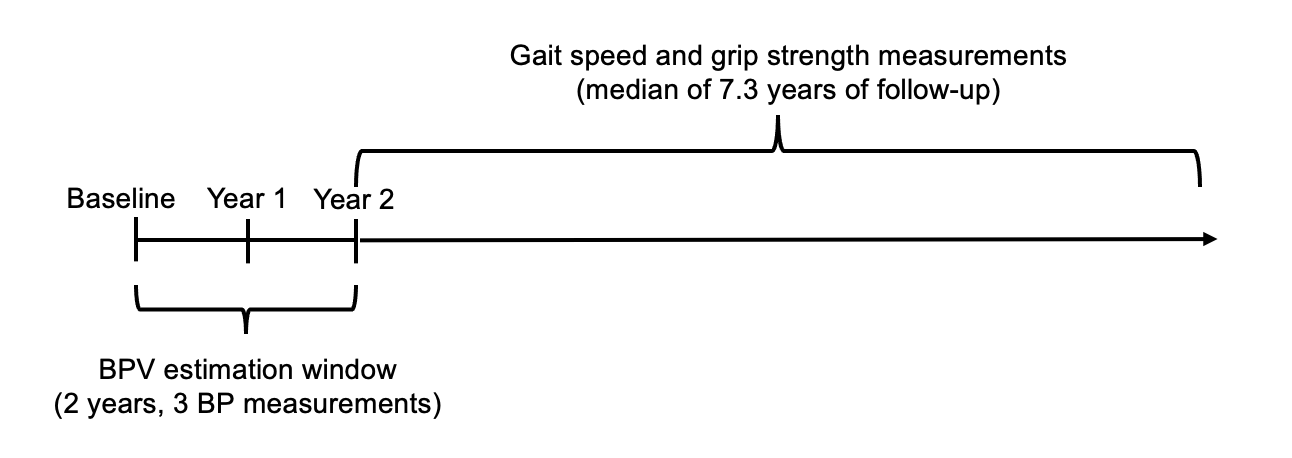
**

**Supplemental Figure 3.** Predicted gait speed and grip strength from fully adjusted model, by gender and BPV tertile. (A) Gait Speed, (B) Grip Strength.

**A.**


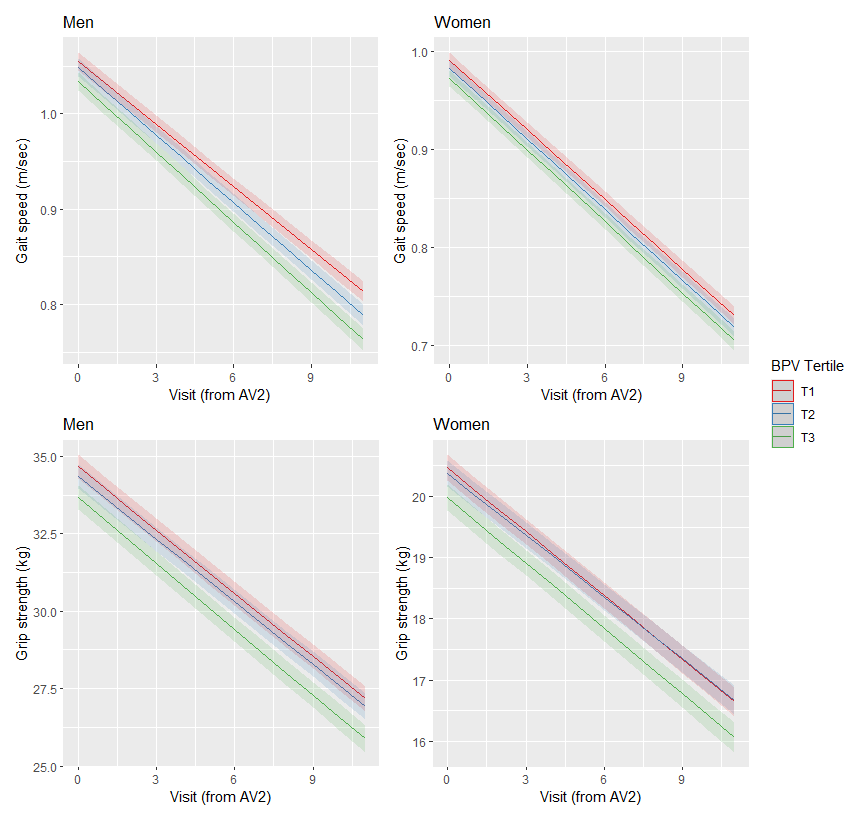

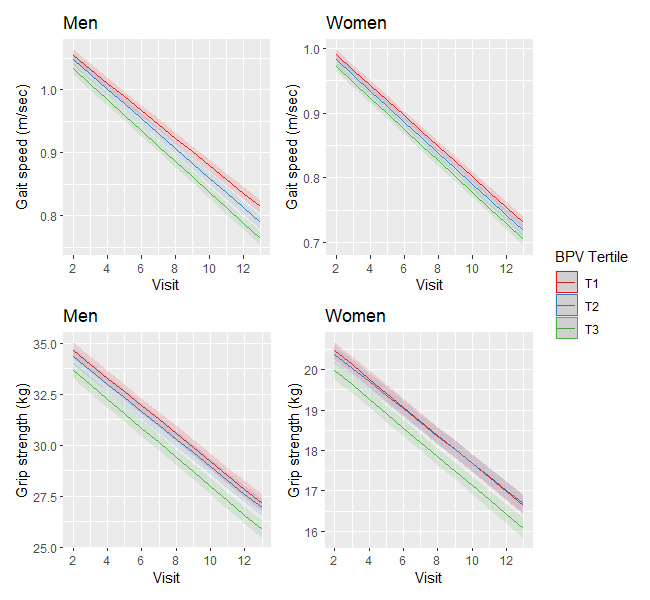


**B.**


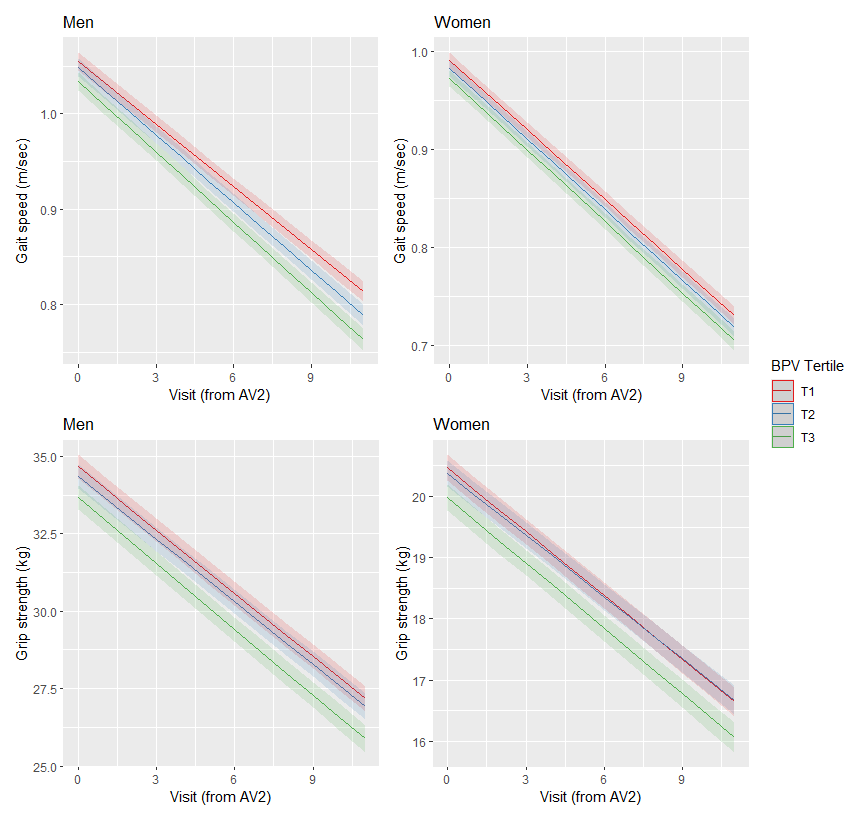
**
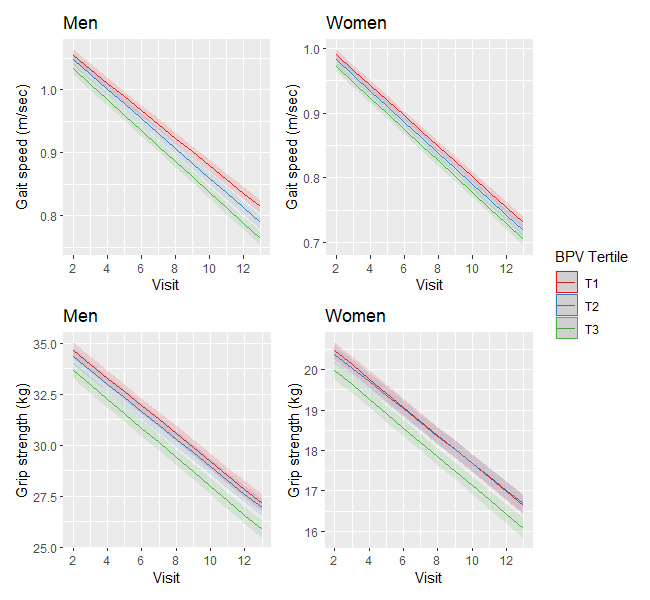
**

**Supplemental Table 1.** Longitudinal changes in gait speed, by gender, with BPV calculated using diastolic blood pressure.

|  | Continuous BPV (per 5mmHg) | | |  | T1 | T2 | | T3 | |
| --- | --- | --- | --- | --- | --- | --- | --- | --- | --- |
| Men, N | 7,376 | | |  | 2,541 | 2,418 | | 2,417 | |
| BPV range |  | | |  | 0-4.0 | 4.2-7.0 | | 7.0-27.2 | |
|  | Time | BPV¹ | Interaction² | Time |  | BPV¹ | Interaction² | BPV¹ | Interaction² |
| Unadjusted | -0.021  (p < 0.001) | -0.014  (p < 0.001) | -0.001  (p < 0.001) | -0.022  (p < 0.001) | Ref. | -0.003  (p = 0.57) | -0.001  (p = 0.01) | -0.022  (p < 0.001) | -0.003  (p < 0.001) |
| Adjusted^3^ | -0.022  (p < 0.001) | -0.005  (p = 0.09) | -0.001  (p < 0.001) | -0.022  (p < 0.001) | Ref. | 0  (p = 0.92) | -0.001  (p = 0.01) | -0.010  (p = 0.05) | -0.003  (p < 0.001) |
| Women, N | 9,311 | | |  | 3,214 | 3,076 | | 3,021 | |
| BPV range |  | | |  | 0-4.4 | 4.5-7.5 | | 7.5-30.0 | |
|  | Time | BPV¹ | Interaction² | Time |  | BPV¹ | Interaction² | BPV¹ | Interaction² |
| Unadjusted | -0.024  (p < 0.001) | -0.019  (p < 0.001) | 0  (p = 0.92) | -0.024  (p < 0.001) | Ref. | -0.014  (p = 0.01) | 0  (p = 0.68) | -0.030  (p < 0.001) | 0.001  (p = 0.27) |
| Adjusted^3^ | -0.024  (p < 0.001) | -0.011  (p < 0.001) | 0  (p = 0.95) | -0.024  (p < 0.001) | Ref. | -0.009  (p = 0.04) | 0  (p = 0.71) | -0.018  (p < 0.001) | 0.001  (p = 0.30) |

¹Estimate of the cross-sectional association of BPV with gait speed at Y2 (BPV was assessed from baseline to Y2, associations of BPV with gait speed are assessed from Y2 to end of follow-up for each participant).

²Time-BPV interaction term to test variation of the gait speed trajectory among BPV levels.

^3^Adjusted for baseline age, race/ethnicity, education, height, abdominal circumference, diabetes, depression, statin use, smoking status, dyslipidemia, and 3MS score; randomized aspirin; and average SBP baseline-Y2

**Supplemental Table 2.** Longitudinal changes in grip strength, by gender, with BPV calculated using diastolic blood pressure.

|  | Continuous BPV (per 5mmHg) | | |  | T1 | T2 | | T3 | |
| --- | --- | --- | --- | --- | --- | --- | --- | --- | --- |
| Men, N | 7,376 | | |  | 2,541 | 2,418 | | 2,417 | |
| BPV range |  | | |  | 0-4.0 | 4.2-7.0 | | 7.0-27.2 | |
|  | Time | BPV¹ | Interaction² | Time |  | BPV¹ | Interaction² | BPV¹ | Interaction² |
| Unadjusted | -0.690  (p < 0.001) | -0.807  (p < 0.001) | 0.007  (p = 0.47) | -0.677  (p < 0.001) | Ref. | -0.369  (p = 0.10) | -0.025  (p = 0.13) | -1.284  (p < 0.001) | 0.009  (p = 0.59) |
| Adjusted^3^ | -0.695  (p < 0.001) | -0.546  (p < 0.001) | 0.007  (p = 0.52) | -0.682  (p < 0.001) | Ref. | -0.278  (p = 0.17) | -0.024  (p = 0.14) | -0.894  (p < 0.001) | 0.008  (p = 0.64) |
| Women, N | 9,311 | | |  | 3,214 | 3,076 | | 3,021 | |
| BPV range |  | | |  | 0-4.4 | 4.5-7.5 | | 7.5-30.0 | |
|  | Time | BPV¹ | Interaction² | Time |  | BPV¹ | Interaction² | BPV¹ | Interaction² |
| Unadjusted | -0.334  (p < 0.001) | -0.312  (p < 0.001) | -0.006  (p = 0.31) | -0.340  (p < 0.001) | Ref. | -0.241  (p = 0.06) | 0.007  (p = 0.53) | -0.496  (p < 0.001) | -0.013  (p = 0.25) |
| Adjusted^3^ | -0.338  (p < 0.001) | -0.185  (p = 0.007) | -0.007  (p = 0.27) | -0.344  (p < 0.001) | Ref. | -0.149  (p = 0.21) | 0.007  (p = 0.54) | -0.290  (p = 0.02) | -0.013  (p = 0.23) |

¹Estimate of the cross-sectional association of BPV with grip strength at Y2 (BPV was assessed from baseline to Y2, associations of BPV with grip strength are assessed from Y2 to end of follow-up for each participant).

²Time-BPV interaction term to test variation of the grip strength trajectory among BPV levels.

^3^Adjusted for baseline age, race/ethnicity, education, height, abdominal circumference, diabetes, depression, statin use, smoking status, dyslipidemia, and 3MS score; randomized aspirin; and average SBP baseline-Y2

**Supplemental Table 3.** Longitudinal changes in gait speed, by gender, with BPV calculated using ARV (average real variability).

|  | Continuous BPV (per 5mmHg) | | |  | T1 | T2 | | T3 | |
| --- | --- | --- | --- | --- | --- | --- | --- | --- | --- |
| Men, N | 7,376 | | |  | 2,642 | 2,341 | | 2,393 | |
| BPV range |  | | |  | 0-8.0 | 8.5-14.0 | | 14.5-67.5 | |
|  | Time | BPV¹ | Interaction² | Time |  | BPV¹ | Interaction² | BPV¹ | Interaction² |
| Unadjusted | -0.021 (p < 0.001) | -0.010 (p < 0.001) | -0.001 (p < 0.001) | -0.022 (p < 0.001) | Ref. | -0.022 (p < 0.001) | -0.001 (p = 0.04) | -0.041 (p < 0.001) | -0.003 (p < 0.001) |
| Adjusted^3^ | -0.022 (p < 0.001) | -0.005 (p < 0.001) | -0.001 (p < 0.001) | -0.022 (p < 0.001) | Ref. | -0.013 (p = 0.008) | -0.001 (p = 0.03) | -0.022 (p < 0.001) | -0.003 (p < 0.001) |
| Women, N | 9,316 | | |  | 3,337 | 3,009 | | 2,970 | |
| BPV range |  | | |  | 0-8.5 | 9.0-15.0 | | 15.5-82.0 | |
|  | Time | BPV¹ | Interaction² | Time |  | BPV¹ | Interaction² | BPV¹ | Interaction² |
| Unadjusted | -0.024 (p < 0.001) | -0.011 (p < 0.001) | 0 (p = 0.652) | -0.024 (p < 0.001) | Ref. | -0.013 (p = 0.01) | 0 (p = 0.92) | -0.039 (p < 0.001) | 0 (p = 0.61) |
| Adjusted^3^ | -0.024 (p < 0.001) | -0.005 (p < 0.001) | 0 (p = 0.492) | -0.024 (p < 0.001) | Ref. | -0.005 (p = 0.22) | 0 (p = 0.97) | -0.017 (p < 0.001) | 0 (p = 0.50) |

¹Estimate of the cross-sectional association of BPV with gait speed at Y2 (BPV was assessed from baseline to Y2, associations of BPV with grip strength are assessed from Y2 to end of follow-up for each participant).

²Time-BPV interaction term to test variation of the gait speed trajectory among BPV levels.

^3^Adjusted for age, race/ethnicity, education, average SBP baseline-Y2, height, abdominal circumference, diabetes, depression, statin use, smoking status, dyslipidemia, 3MS, randomized aspirin

**Supplemental Table 4.** Longitudinal changes in grip strength, by gender, with BPV calculated using ARV (average real variability).

|  | Continuous BPV (per 5mmHg) | | |  | T1 | T2 | | T3 | |
| --- | --- | --- | --- | --- | --- | --- | --- | --- | --- |
| Men, N | 7,376 | | |  | 2,642 | 2,341 | | 2,393 | |
| BPV range |  | | |  | 0-8.0 | 8.5-14.0 | | 14.5-67.5 | |
|  | Time | BPV¹ | Interaction² | Time |  | BPV¹ | Interaction² | BPV¹ | Interaction² |
| Unadjusted | -0.657 (p < 0.001) | -0.398 (p < 0.001) | -0.011 (p = 0.02) | -0.676 (p < 0.001) | Ref. | -0.861 (p < 0.001) | 0.011 (p = 0.48) | -1.641 (p < 0.001) | -0.033 (p = 0.04) |
| Adjusted^3^ | -0.661 (p < 0.001) | -0.225 (p < 0.001) | -0.011 (p = 0.01) | -0.680 (p < 0.001) | Ref. | -0.511 (p = 0.01) | 0.010 (p = 0.53) | -0.977 (p < 0.001) | -0.036 (p = 0.03) |
| Women, N | 9,316 | | |  | 3,337 | 3,009 | | 2,970 | |
| BPV range |  | | |  | 0-8.5 | 9.0-15.0 | | 15.5-82.0 | |
|  | Time | BPV¹ | Interaction² | Time |  | BPV¹ | Interaction² | BPV¹ | Interaction² |
| Unadjusted | -0.338 (p < 0.001) | -0.201 (p < 0.001) | -0.002 (p = 0.51) | -0.339 (p < 0.001) | Ref. | -0.155 (p = 0.22) | 0 (p = 0.99) | -0.681 (p < 0.001) | -0.011 (p = 0.31) |
| Adjusted^3^ | -0.341 (p < 0.001) | -0.115 (p < 0.001) | -0.002 (p = 0.42) | -0.343 (p < 0.001) | Ref. | -0.101 (p = 0.40) | 0.001 (p = 0.94) | -0.378 (p = 0.002) | -0.012 (p = 0.27) |

¹Estimate of the cross-sectional association of BPV with grip strength at Y2 (BPV was assessed from baseline to Y2, associations of BPV with grip strength are assessed from Y2 to end of follow-up for each participant).

²Time-BPV interaction term to test variation of the grip strength trajectory among BPV levels.

^3^Adjusted for age, race/ethnicity, education, average SBP baseline-Y2, height, abdominal circumference, diabetes, depression, statin use, smoking status, dyslipidemia, 3MS, randomized aspirin

**Supplemental Table 5.** Longitudinal changes in gait speed, by gender, excluding participants who reported use of a walking aid at baseline.

|  | Continuous BPV (per 5mmHg) | | |  | T1 | T2 | | T3 | |
| --- | --- | --- | --- | --- | --- | --- | --- | --- | --- |
| Men, N | 7,272 | | |  | 2,483 | 2,381 | | 2,408 | |
| BPV range |  | | |  | 0-6.66 | 6.81-11.53 | | 11.55-41.74 | |
|  | Time | BPV¹ | Interaction² | Time |  | BPV¹ | Interaction² | BPV¹ | Interaction² |
| Unadjusted | -0.021 (p < 0.001) | -0.016 (p < 0.001) | -0.001 (p < 0.001) | -0.022 (p < 0.001) | Ref. | -0.015 (p = 0.009) | -0.002 (p = 0.006) | -0.039 (p < 0.001) | -0.003 (p < 0.001) |
| Adjusted^3^ | -0.021 (p < 0.001) | -0.008 (p < 0.001) | -0.001 (p < 0.001) | -0.022 (p < 0.001) | Ref. | -0.006 (p = 0.23) | -0.002 (p = 0.005) | -0.020 (p < 0.001) | -0.003 (p < 0.001) |
| Women, N | 9,066 | | |  | 3,059 | 3,012 | | 2,995 | |
| BPV range |  | | |  | 0-7.02 | 7.09-12.06 | | 12.10-57.71 | |
|  | Time | BPV¹ | Interaction² | Time |  | BPV¹ | Interaction² | BPV¹ | Interaction² |
| Unadjusted | -0.024 (p < 0.001) | -0.014 (p < 0.001) | 0 (p = 0.45) | -0.023 (p < 0.001) | Ref. | -0.012 (p = 0.03) | -0.001 (p = 0.30) | -0.036 (p < 0.001) | -0.001 (p = 0.20) |
| Adjusted^3^ | -0.024 (p < 0.001) | -0.006 (p < 0.001) | 0 (p = 0.30) | -0.024 (p < 0.001) | Ref. | -0.007 (p = 0.12) | 0 (p = 0.41) | -0.017 (p < 0.001) | -0.001 (p = 0.15) |

¹Estimate of the cross-sectional association of BPV with gait speed at Y2 (BPV was assessed from baseline to Y2, associations of BPV with gait speed are assessed from Y2 to end of follow-up for each participant).

²Time-BPV interaction term to test variation of the gait speed trajectory among BPV levels.

^3^Adjusted for age, race/ethnicity, education, average SBP baseline-Y2, height, abdominal circumference, diabetes, depression, statin use, smoking status, dyslipidemia, 3MS, randomized aspirin

**Supplemental Table 6.** Longitudinal changes in gait speed, by gender, with long-term blood pressure variability calculated using four blood pressure measurements (from baseline to the year 3 visit)^1^.

|  | Continuous BPV (per 5mmHg) | | |  | T1 | T2 | | T3 | |
| --- | --- | --- | --- | --- | --- | --- | --- | --- | --- |
| Men, N | 6,665 | | |  | 2,229 | 2,220 | | 2,216 | |
| BPV range |  | | |  | 0.82-7.59 | 7.59-11.95 | | 11.96-34.09 | |
|  | Time | BPV^2^ | Interaction^3^ | Time |  | BPV^2^ | Interaction^3^ | BPV^2^ | Interaction^3^ |
| Unadjusted | -0.022 (p < 0.001) | -0.020 (p < 0.001) | -0.001 (p < 0.001) | -0.024 (p < 0.001) | Ref. | -0.018 (p = 0.003) | -0.001 (p = 0.08) | -0.047 (p < 0.001) | -0.002 (p = 0.02) |
| Adjusted^4^ | -0.023 (p < 0.001) | -0.011 (p < 0.001) | -0.001 (p < 0.001) | -0.024 (p < 0.001) | Ref. | -0.010 (p = 0.07) | -0.001 (p = 0.06) | -0.028 (p < 0.001) | -0.002 (p = 0.02) |
| Women, N | 8,431 | | |  | 2,830 | 2,793 | | 2,808 | |
| BPV range |  | | |  | 0.50-8.04 | 8.06-12.40 | | 12.41-48.00 | |
|  | Time | BPV^2^ | Interaction^3^ | Time |  | BPV^2^ | Interaction^3^ | BPV^2^ | Interaction^3^ |
| Unadjusted | -0.024 (p < 0.001) | -0.018 (p < 0.001) | 0 (p = 0.13) | -0.024 (p < 0.001) | Ref. | -0.017 (p = 0.004) | -0.001 (p = 0.41) | -0.045 (p < 0.001) | -0.001 (p = 0.15) |
| Adjusted^4^ | -0.024 (p < 0.001) | -0.007 (p < 0.001) | 0 (p = 0.07) | -0.025 (p < 0.001) | Ref. | -0.007 (p = 0.14) | 0 (p = 0.54) | -0.017 (p < 0.001) | -0.001 (p = 0.11) |

^1^BPV was assessed using blood pressure measurements from baseline to the year 2 annual visit in the main analyses.

^2^Estimate of the cross-sectional association of BPV with gait speed at Y3 (BPV was assessed from baseline to Y3, associations of BPV with gait speed are assessed from Y3 to end of follow-up for each participant).

^3^Time-BPV interaction term to test variation of the gait speed trajectory among BPV levels.

^4^Adjusted for age, race/ethnicity, education, average SBP baseline-Y2, height, abdominal circumference, diabetes, depression, statin use, smoking status, dyslipidemia, 3MS, randomized aspirin

**Supplemental Table 7.** Longitudinal changes in grip strength, by gender, with long-term blood pressure variability calculated using four blood pressure measurements (from baseline to the year 3 visit)^1^.

|  | Continuous BPV (per 5mmHg) | | |  | T1 | T2 | | T3 | |
| --- | --- | --- | --- | --- | --- | --- | --- | --- | --- |
| Men, N | 6,677 | | |  | 2,231 | 2,223 | | 2,223 | |
| BPV range |  | | |  | 0.82-7.59 | 7.59-11.95 | | 11.96-34.09 | |
|  | Time | BPV^2^ | Interaction^3^ | Time |  | BPV^2^ | Interaction^3^ | BPV^2^ | Interaction^3^ |
| Unadjusted | -0.664 (p < 0.001) | -0.736 (p < 0.001) | -0.014 (p = 0.11) | -0.693 (p < 0.001) | Ref. | -1.069 (p < 0.001) | 0.028 (p = 0.17) | -1.684 (p < 0.001) | -0.026 (p = 0.23) |
| Adjusted^4^ | -0.669 (p < 0.001) | -0.428 (p < 0.001) | -0.014 (p = 0.10) | -0.699 (p < 0.001) | Ref. | -0.779 (p < 0.001) | 0.027 (p = 0.19) | -1.028 (p < 0.001) | -0.027 (p = 0.20) |
| Women, N | 8,455 | | |  | 2,827 | 2,815 | | 2,813 | |
| BPV range |  | | |  | 0.50-8.04 | 8.06-12.45 | | 12.45-48.00 | |
|  | Time | BPV^2^ | Interaction^3^ | Time |  | BPV^2^ | Interaction^3^ | BPV^2^ | Interaction^3^ |
| Unadjusted | -0.328 (p < 0.001) | -0.390 (p < 0.001) | -0.005 (p = 0.32) | -0.342 (p < 0.001) | Ref. | -0.144 (p = 0.30) | 0.010 (p = 0.47) | -0.856 (p < 0.001) | -0.003 (p = 0.83) |
| Adjusted^4^ | -0.331 (p < 0.001) | -0.213 (p < 0.001) | -0.006 (p = 0.25) | -0.347 (p < 0.001) | Ref. | -0.011 (p = 0.93) | 0.011 (p = 0.43) | -0.428 (p = 0.001) | -0.005 (p = 0.71) |

^1^BPV was assessed using blood pressure measurements from baseline to the year 2 annual visit in the main analyses.

^2^Estimate of the cross-sectional association of BPV with grip strength at Y3 (BPV was assessed from baseline to Y3, associations of BPV with grip strength are assessed from Y3 to end of follow-up for each participant).

^3^Time-BPV interaction term to test variation of the grip strength trajectory among BPV levels.

^4^Adjusted for age, race/ethnicity, education, average SBP baseline-Y2, height, abdominal circumference, diabetes, depression, statin use, smoking status, dyslipidemia, 3MS, randomized aspirin

**Supplemental Table 8.** Longitudinal changes in gait speed, by gender and hypertension^1^ status at baseline.

|  | Continuous BPV (per 5mmHg) | | |  | T1 | T2 | | T3 | |
| --- | --- | --- | --- | --- | --- | --- | --- | --- | --- |
| Men |  | | |  |  |  | |  | |
|  | Time | BPV^2^ | Interaction^3^ | Time |  | BPV^2^ | Interaction^3^ | BPV^2^ | Interaction^3^ |
| *Normotensive, N* | 1,848 | | |  | 621 | 615 | | 612 | |
| *BPV range* |  |  |  |  | 0-5.6 | 5.7-9.6 | | 9.7-31.0 | |
| Unadjusted | -0.020  (p < 0.001) | -0.016  (p = 0.001) | 0  (p = 0.53) | -0.021  (p < 0.001) | Ref. | -0.025  (p = 0.02) | 0.001  (p = 0.46) | -0.037  (p < 0.001) | 0  (p = 0.88) |
| Adjusted^4^ | -0.020  (p < 0.001) | -0.009  (p = 0.06) | 0  (p = 0.48) | -0.021  (p < 0.001) | Ref. | -0.019  (p = 0.07) | 0.001  (p = 0.42) | -0.021  (p = 0.04) | 0  (p = 0.94) |
| *Hypertensive, N* |  | 5,528 |  |  | 1,852 | 1,840 | | 1,836 | |
| *BPV range* |  |  |  |  | 0-7.0 | 7.1-12.2 | | 12.3-41.7 | |
| Unadjusted | -0.022  (p < 0.001) | -0.015  (p < 0.001) | -0.001  (p < 0.001) | -0.022  (p < 0.001) | Ref. | -0.013  (p = 0.05) | -0.003  (p < 0.001) | -0.043  (p < 0.001) | -0.003  (p < 0.001) |
| Adjusted^4^ | -0.022  (p < 0.001) | -0.008  (p < 0.001) | -0.001  (p < 0.001) | -0.022  (p < 0.001) | Ref. | -0.007  (p = 0.24) | -0.003  (p < 0.001) | -0.025  (p < 0.001) | -0.003  (p < 0.001) |
| Women |  | | |  |  |  | |  | |
|  | Time | BPV¹ | Interaction² | Time |  | BPV¹ | Interaction² | BPV¹ | Interaction² |
| *Normotensive, N* | 2,516 | | |  | 858 | 820 | | 838 | |
| *BPV range* |  |  |  |  | 0-5.7 | 5.8-9.6 | | 9.6-37.5 | |
| Unadjusted | -0.021  (p < 0.001) | -0.014  (p < 0.001) | -0.001  (p = 0.004) | -0.022  (p < 0.001) | Ref. | 0.001  (p = 0.94) | -0.001  (p = 0.42) | -0.026  (p = 0.01) | -0.001  (p = 0.15) |
| Adjusted^4^ | -0.021  (p < 0.001) | -0.005  (p = 0.21) | -0.001  (p = 0.002) | -0.022  (p < 0.001) | Ref. | 0.005  (p = 0.58) | -0.001  (p = 0.40) | -0.006  (p = 0.50) | -0.002  (p = 0.10) |
| *Hypertensive, N* |  | 6,800 |  |  | 2,280 | 2,267 | | 2,253 | |
| *BPV range* |  |  |  |  | 0-7.6 | 7.6-12.9 | | 12.9-57.7 | |
| Unadjusted | -0.025  (p < 0.001) | -0.012  (p < 0.001) | 0  (p = 0.13) | -0.024  (p < 0.001) | Ref. | -0.008  (p = 0.19) | 0  (p = 0.89) | -0.030  (p < 0.001) | 0.001  (p = 0.38) |
| Adjusted^4^ | -0.025  (p < 0.001) | -0.007  (p < 0.001) | 0  (p = 0.20) | -0.025  (p < 0.001) | Ref. | -0.011  (p = 0.03) | 0  (p = 0.75) | -0.021  (p < 0.001) | 0  (p = 0.49) |

^1^Hypertension was defined as a blood pressure of >140/90 or use of an antihypertensive medication at baseline.

^2^Estimate of the cross-sectional association of BPV with gait speed at Y2 (BPV was assessed from baseline to Y2, associations of BPV with gait speed are assessed from Y2 to end of follow-up for each participant).

^3^Time-BPV interaction term to test variation of the gait speed trajectory among BPV levels.

^4^Adjusted for baseline age, race/ethnicity, education, height, abdominal circumference, diabetes, depression, statin use, smoking status, dyslipidemia, and 3MS score; randomized aspirin; and average SBP baseline-Y2

**Supplemental Table 9.** Longitudinal changes in grip strength, by gender and hypertension^1^ status at baseline.

|  | Continuous BPV (per 5mmHg) | | |  | T1 | T2 | | T3 | |
| --- | --- | --- | --- | --- | --- | --- | --- | --- | --- |
| Men |  | | |  |  |  | |  | |
|  | Time | BPV^2^ | Interaction^3^ | Time |  | BPV^2^ | Interaction^3^ | BPV^2^ | Interaction^3^ |
| *Normotensive, N* |  | 1,848 |  |  | 621 | 615 | | 612 | |
| *BPV range* |  |  |  |  | 0-5.6 | 5.7-9.6 | | 9.7-31.0 | |
| Unadjusted | -0.619  (p < 0.001) | -0.645  (p < 0.001) | -0.011  (p = 0.42) | -0.658  (p < 0.001) | Ref. | -0.820  (p = 0.06) | 0.073  (p = 0.02) | -1.469  (p < 0.001) | -0.015  (p = 0.64) |
| Adjusted^4^ | -0.622  (p < 0.001) | -0.519  (p = 0.003) | -0.012  (p = 0.38) | -0.662  (p < 0.001) | Ref. | -0.693  (p = 0.09) | 0.074  (p = 0.02) | -1.047  (p = 0.01) | -0.017  (p = 0.58) |
| *Hypertensive, N* |  | 5,528 |  |  | 1,852 | 1,840 | | 1,836 | |
| *BPV range* |  |  |  |  | 0-7.0 | 7.1-12.2 | | 12.3-41.7 | |
| Unadjusted | -0.673  (p < 0.001) | -0.628  (p < 0.001) | -0.012  (p = 0.07) | -0.684  (p < 0.001) | Ref. | -0.699  (p = 0.01) | -0.012  (p = 0.53) | -1.698  (p < 0.001) | -0.033  (p = 0.09) |
| Adjusted^4^ | -0.677  (p < 0.001) | -0.362  (p < 0.001) | -0.013  (p = 0.05) | -0.688  (p < 0.001) | Ref. | -0.442  (p = 0.06) | -0.013  (p = 0.49) | -1.004  (p < 0.001) | -0.035  (p = 0.07) |
| Women |  | | |  |  |  | |  | |
|  | Time | BPV¹ | Interaction² | Time |  | BPV¹ | Interaction² | BPV¹ | Interaction² |
| *Normotensive, N* |  | 2,516 |  |  | 858 | 820 | | 838 | |
| *BPV range* |  |  |  |  | 0-5.7 | 5.8-9.6 | | 9.6-37.5 | |
| Unadjusted | -0.298  (p < 0.001) | -0.295  (p = 0.003) | -0.005  (p = 0.56) | -0.308  (p < 0.001) | Ref. | -0.145  (p = 0.55) | -0.002  (p = 0.90) | -0.675  (p = 0.005) | 0.009  (p = 0.64) |
| Adjusted^4^ | -0.300  (p < 0.001) | -0.147  (p = 0.13) | -0.006  (p = 0.50) | -0.311  (p < 0.001) | Ref. | -0.061  (p = 0.79) | -0.001  (p = 0.96) | -0.352  (p = 0.13) | 0.008  (p = 0.68) |
| *Hypertensive, N* |  | 6,800 |  |  | 2,280 | 2,267 | | 2,253 | |
| *BPV range* |  |  |  |  | 0-7.6 | 7.6-12.9 | | 12.9-57.7 | |
| Unadjusted | -0.362  (p < 0.001) | -0.242  (p < 0.001) | 0.002  (p = 0.63) | -0.355  (p < 0.001) | Ref. | 0.115  (p = 0.45) | -0.002  (p = 0.86) | -0.646  (p < 0.001) | -0.005  (p = 0.72) |
| Adjusted^4^ | -0.366  (p < 0.001) | -0.166  (p < 0.001) | 0.002  (p = 0.66) | -0.359  (p < 0.001) | Ref. | 0.119  (p = 0.40) | -0.002  (p = 0.89) | -0.467  (p = 0.001) | -0.005  (p = 0.68) |

^1^Hypertension was defined as a blood pressure of >140/90 or use of an antihypertensive medication at baseline.

^2^Estimate of the cross-sectional association of BPV with grip strength at Y2 (BPV was assessed from baseline to Y2, associations of BPV with grip strength are assessed from Y2 to end of follow-up for each participant).

^3^Time-BPV interaction term to test variation of the grip strength trajectory among BPV levels.

^4^Adjusted for baseline age, race/ethnicity, education, height, abdominal circumference, diabetes, depression, statin use, smoking status, dyslipidemia, and 3MS score; randomized aspirin; and average SBP baseline-Y2
